# A conformational selection mechanism of flavivirus NS5 for species-specific STAT2 inhibition

**DOI:** 10.1038/s42003-024-05768-8

**Published:** 2024-01-10

**Authors:** Mahamaya Biswal, Wangyuan Yao, Jiuwei Lu, Jianbin Chen, Juliet Morrison, Rong Hai, Jikui Song

**Affiliations:** 1 Department of Biochemistry, University of California, Riverside, CA USA; 2 Department of Microbiology and Plant Pathology, University of California, Riverside, CA USA

**Keywords:** Cryoelectron microscopy, Virus-host interactions

## Abstract

Flaviviruses, including Zika virus (ZIKV) and Dengue virus (DENV), rely on their non-structural protein 5 (NS5) for both replication of viral genome and suppression of host IFN signaling. DENV and ZIKV NS5s were shown to facilitate proteosome-mediated protein degradation of human STAT2 (hSTAT2). However, how flavivirus NS5s have evolved for species-specific IFN-suppression remains unclear. Here we report structure-function characterization of the DENV serotype 2 (DENV2) NS5−hSTAT2 complex. The MTase and RdRP domains of DENV2 NS5 form an extended conformation to interact with the coiled-coil and N-terminal domains of hSTAT2, thereby promoting hSTAT2 degradation in cells. Disruption of the extended conformation of DENV2/ZIKV NS5, but not the alternative compact state, impaired their hSTAT2 binding. Our comparative structural analysis of flavivirus NS5s further reveals a conserved protein-interaction platform with subtle amino-acid variations likely underpinning diverse IFN-suppression mechanisms. Together, this study uncovers a conformational selection mechanism underlying species-specific hSTAT2 inhibition by flavivirus NS5.

## Abstract (web summary)

The dengue viral protein NS5 interacts with human STAT2 in a conformational selection mechanism, and structural analysis reveals that subtle amino acid variation in NS5 proteins underpins their species-specific host interactions.

## Introduction

Flaviviruses belong to the Flaviviridae family with a positive, single-strand RNA genome^1^. Some of the flaviviruses, such as Dengue virus (DENV), Zika virus (ZIKV), West Niles virus (WNV), and Yellow Fever virus (YFV), are medically relevant pathogens and pose a threat to public health globally^2^. Of particular note, global incidence of DENV infection is continuously growing with ~400 million cases of infection and ~20 thousand deaths per year^3^. In addition, the outbreak of ZIKV during 2015–2017 caused tens of thousands of infections across the Americas, creating a worldwide heath crisis^4^. These everlasting threats of flaviviruses raise an urgent need to development of therapeutic agents against their infection.

Flavivirus genome is ~10 kb in size, encoding three structural proteins (M, S and P) for capsid formation and seven non-structural proteins (NS1, NS2, NS3a, NS3b, NS4a, NS4b, and NS5) for genome replication. Among these, the non-structural protein 5 (NS5) is the most conserved viral protein essential for replication of viral genome^5,6^. In addition, all flavivirus NS5 proteins examined so far serve as antagonist for type-I interferon (IFN-I) signaling pathway to support viral infection^7–14^. NS5 protein is comprised of two domains: an N-terminal methyltransferase (MTase) domain responsible for RNA capping to protect the viral genome and a C-terminal RNA-dependent RNA polymerase (RdRP) domain for replicating the viral genome^15^. Whereas the MTase and RdRP domains were each structurally conserved, two alternative domain conformations of flavivirus NS5 have been observed: the compact conformation by DENV serotype 3 (DENV3) NS5^16^ and the extended conformation by the NS5 proteins from Japanese encephalitis virus (JEV), YFV and ZIKV^17–21^. Both conformations were observed for DENV2 NS5^22,23^, supporting the notion that the two conformations coexist in solution^16,18–21,24^. The functional context of these two alternative conformations remains unclear. Nevertheless, recent studies indicated that both conformations are required for DENV NS5-mediated replication^23,25^.

The IFN-I signaling is triggered by cellular receptors recognizing the Pathogen Associated Molecular Pattern (PAMP) expressed by viruses^26^. Subsequently, IFN-I binds to the IFNAR receptor to activate Janus kinases - JAK1 and TYK2, which in turn phosphorylate signal transducers of transcription 2 (STAT2) and further recruit 1 (STAT1) to form a heterodimer. The heterodimer of STAT1 and STAT2 then associates with IFN regulatory factor 9 (IRF9) to form the IFN-stimulated gene factor-3 (ISGF3) complex, which binds to IFN-stimulated responsive elements (ISREs) in the nucleus to stimulate the transcription of interferon-stimulated genes (ISGs)^27–30^. To support viral infection, flavivirus NS5s have evolved with diverse mechanisms to suppress IFN signaling^7–14^. For instance, DENV NS5 interacts with human STAT2 (hSTAT2) to promote protein degradation of hSTAT2 mediated by ubiquitin ligase UBR4^7,14^. ZIKV NS5, but not its closer relative Spondweni virus (SPOV) NS5, shares a similar mechanism of hSTAT2 inhibition with DENV NS5, although in a UBR4-indpeendent manner^9^. In addition, YFV NS5 interacts with hSTAT2 only upon IFN stimulation^11^. In contrast, many flaviviruses (e.g. WNV, JEV and SPOV) employed theirs NS5s to suppress other players of IFN signaling^8–13^. How flavivirus NS5 proteins have evolved into species-specific IFN-antagonizing functionalities is currently unclear.

To elaborate the molecular basis for the interaction between ZIKV NS5 and hSTAT2, we recently reported the crystal structure of the RdRP domain of ZIKV NS5 in complex with an N-terminal fragment of hSTAT2 (residues 1-713), as well as the cryo-EM structure of full-length ZIKV NS5 and hSTAT2^31^. The crystal structure of the ZIKV RdRP−hSTAT2 complex reveals that the NS5 RdRP domain interacts with the coiled-coil domain (CCD) and the N-terminal domain (ND) of hSTAT2, resulting in two discrete interaction interfaces^31^. On the other hand, the cryo-EM structure of the ZIKV NS5−hSTAT2 complex reveals that the hSTAT2 CCD domain is anchored at the MTase−RdRP interdomain cleft, engaging a multivalent interaction with both the MTase and RdRP domains^31^. In contrast to that in the crystal structure of ZIKV RdRP–hSTAT2 complex, the density for the ND domain of hSTAT2 was not identified in the cryo-EM study of ZIKV NS5−hSTAT2 complex, likely due to its modest resolution (4 Å), raising a question whether this domain is indeed involved in the NS5−hSTAT2 interaction in solution.

To gain a comprehensive view on the flavivirus NS5−hSTAT2 interaction, we performed structural, biochemical and cellular analyses of the complex between DENV2 NS5 and hSTAT2. The cryo-EM structure of DENV2 NS5−hSTA2 complex reveals a multivalent interaction involving the MTase and RdRP domains of DENV2 NS5 and the ND and CCD domains of hSTAT2. DENV2 NS5 adopts the extended conformation to harbor the hSTAT2 CCD at the interdomain cleft formed by the DENV2 MTase and RdRP domains, resembling but distinct from what was previously observed for the ZIKV NS5−hSTAT2 complex^31^. In addition, the hSTAT2 ND interacts with DENV2 RdRP at the RNA exit channel. Disruption of the extended conformation, but not the compact conformation, of DENV2 NS5 led to reduced DENV2 NS5−hSTAT2 interaction in vitro and ex vivo, and impaired DENV2 NS5-mediated hSTAT2 degradation. This conformational selection mechanism of the NS5−hSTAT2 interaction was also shared by ZIKV NS5, suggesting that flavivirus NS5 may employ a conserved fashion for functional antagonism of STAT2 or other IFN players. Finally, structural comparison of flavivirus NS5s suggests a common protein-interaction platform, with subtle amino-acid variation likely underpinning their distinct IFN suppression mechanisms. Together, this study provides an evolutionary view on how flavivirus NS5 has evolved for IFN suppression.

## Results

### Structural overview of the DENV2 NS5−hSTAT2 complex

**Fig. 1:**
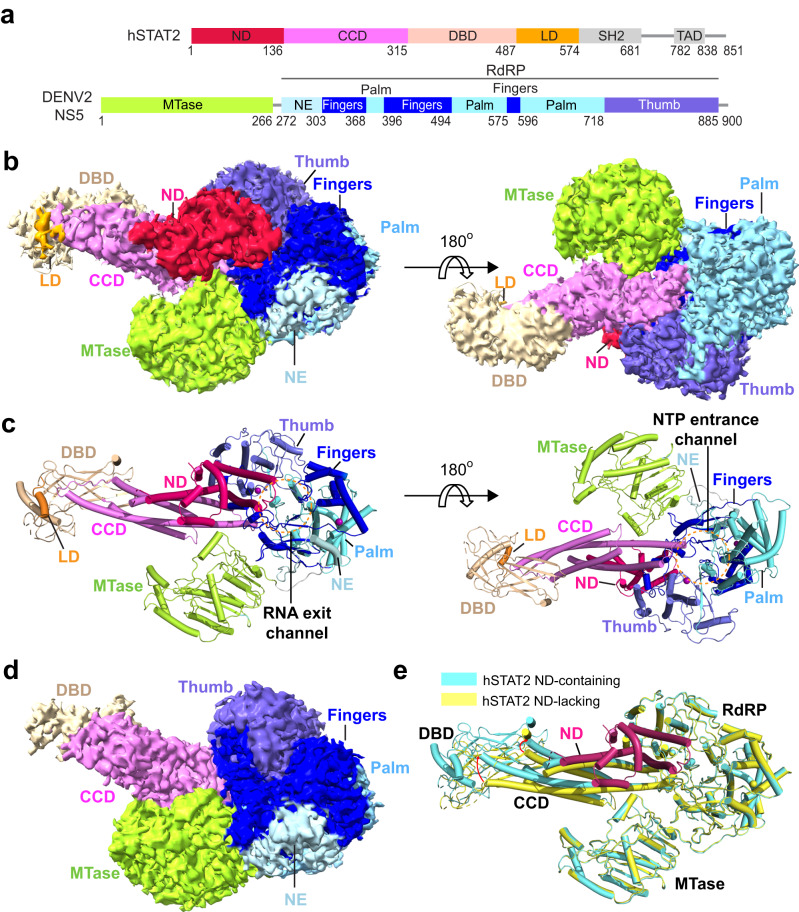
Cryo-EM structure of DENV2 NS5–hSTAT2 complex. **a** Primary sequence of hSTAT2 and DENV2 NS5, with individual domains color coded. **b** Shaded surface views of the cryo-EM density map of the DENV2 NS5–hSTAT2 complex, with the hSTAT2 ND ordered, from opposite sides. **c** Opposite views of the DENV2 NS5–hSTAT2 complex in cartoon representation. **d** Cryo-EM density map of the DENV2 NS5–hSTAT2 complex with the hSTAT2 ND disordered. **e** Structural overlay between the DENV2 NS5–hSTAT2 complex with the hSTAT2 ND disordered and that with the hSTAT2 ND ordered.

To provide the molecular basis for the DENV2 NS5−hSTAT2 interaction, we performed single particle cryo-EM analysis for full-length DENV2 NS5 in complex with full-length hSTAT2 (Fig. 1a). After 3D classification, two classes of particles with pronounced features of DENV2 NS5−hSTAT2 interactions were identified (Supplementary Fig. 1). The first class shows a density of full-length DENV2 NS5 and hSTAT2 but is intractable for structural analysis due to strong orientational bias (Supplementary Fig. 1). The second class permits high-resolution structural characterization, despite coincidently lack of density for the C-terminal linker domain (LD) and the SH2 domain of hSTAT2 (See details below). 3D variability analysis of the second class via cryoSPARC^32,33^ further revealed two sub-classes of particles: the first class was solved with 3.3-Å resolution, containing an ordered N-terminal domain (ND) of hSTAT2 (Fig. 1b, c and Supplementary Fig. 1 and 2a–c and Table 1); the second class was solved with 3.4-Å resolution, lacking an ordered hSTAT2 ND (Fig. 1d and Supplementary Fig. 1 and 2d–f and Table 1). Atomic models were built for both types, revealing nearly identical structures for the regions beyond the hSTAT2 ND, except for a slight tilt of the hSTAT2 CCD and DBD (Fig. 1e). We therefore focused on the class with ordered hSTAT2 ND for structural analysis of the DENV2 NS5−hSTAT2 complex hereinafter.

**Table 1:** Cryo-EM data collection, refinement and validation statistics.

States	DENV2 NS5−hSTAT2 complex with STAT2 ND ordered	DENV2 NS5−hSTAT2 complex with hSTAT2 ND disordered
Codes	(EMD-40952, PDB 8T12)	(EMD-40953, PDB 8T13)
Data collection and processing		
Microscope	Titan Krios	
Camera	K3 BioQuantum	
Magnification	81,000	
Voltage (kV)	300	
Defocus range (μm)	−1.0 to −2.0	
Exposure time (s)	3.6	
Dose rate(*e*^*–*^/Å^2^/s)	13.9	
Number of frames	40	
Pixel size (Å)	1.12	
Micrographs (no.)	3112	
Initial particles (no.)	1,574,562	
Symmetry imposed	*C1*	*C1*
Initial particles (no.)	108,409	92,065
Final particles (no.)	99,938	86,928
Map resolution (Å)	3.4	3.45
FSC threshold	0.143	0.143
Refinement		
Initial model used	6UX2, 6KR2	6UX2,6KR2
Model resolution (Å)	3.4	3.45
FSC threshold	0.5	0.5
Map sharpening *B* factor (Å^2^)	−70	−95
CC (mask)	0.73	0.67
Model composition		
Non-hydrogen atoms	10,813	9304
Protein residues	1343	1167
Ion (zinc)	2	2
* B* factors (Å^2^)		
Protein	183.17	222.48
Ion (zinc)	170.32	85.62
R.m.s. deviations		
Bond lengths (Å)	0.005	0.002
Bond angles (°)	0.999	0.526
Validation		
MolProbity score	1.89	1.73
Clashscore	9.15	7.19
Poor rotamers (%)	0.60	0.1
Ramachandran plot		
Favored (%)	94	95.16
Allowed (%)	5.63	4.84
Disallowed (%)	0.38	0

The cryo-EM map of the DENV2 NS5−hSTAT2 complex permitted us to model both the MTase and RdRP domains of DENV2 NS5, with the former dominated by a Rossmann fold and the latter comprised of the N-terminal extension (NE), Fingers, Palm and Thumb subdomains (Fig. 1b and Supplementary Fig. 3). In addition, we were able to trace the hSTAT2 ND in a helical fold, linked via a disordered linker to the structurally independent core fragment formed by the closely packed CCD in a four-helix bundle, DNA-binding domain (DBD) in an α/β fold, and the N-terminal segment of LD domain (Fig. 1b and Supplementary Fig. 3). The complex of DENV2 NS5−hSTAT2 is assembled into a mushroom shape, with the roof formed by the NS5 MTase and RdRP and the hSTAT2 ND, and the stem formed by hSTAT2 CCD and DBD (Fig. 1b). As mentioned earlier, previous studies have established that DENV2 NS5 adopts at least two alternative conformations in solution: an extended conformation with the MTase and RdRP domains away from each other and a compact conformation with the two domains in close proximity^23^. Here, the MTase and RdRP domains are arranged in a manner resembling the extended conformation, creating an open interdomain cleft that harbors the protruding end of the first two α-helices of the hSTAT2 CCD via surface complementarity (Fig. 1b, c). Notably, the interaction of hSTAT2 CCD with DENV2 RdRP also leads to partial occlusion of the nucleoside triphosphate (NTP) entrance channel of the latter^25^ (Fig. 1c, right). On a distant surface of DENV2 NS5, the hSTAT2 ND is docked next to the RNA exit channel formed by the Fingers of the RdRP, which reinforces the NS5-hSTAT2 interaction (Fig. 1c, left). These data confirm a previous notion that the interaction between flavivirus NS5 and hSTAT2 not only results in inhibition of STAT2-mediated IFN response, but also limits the RdRP activity of the NS5 protein^31^.

### Structural details for the DENV2 NS5−hSTAT2 interaction

**Fig. 2:**
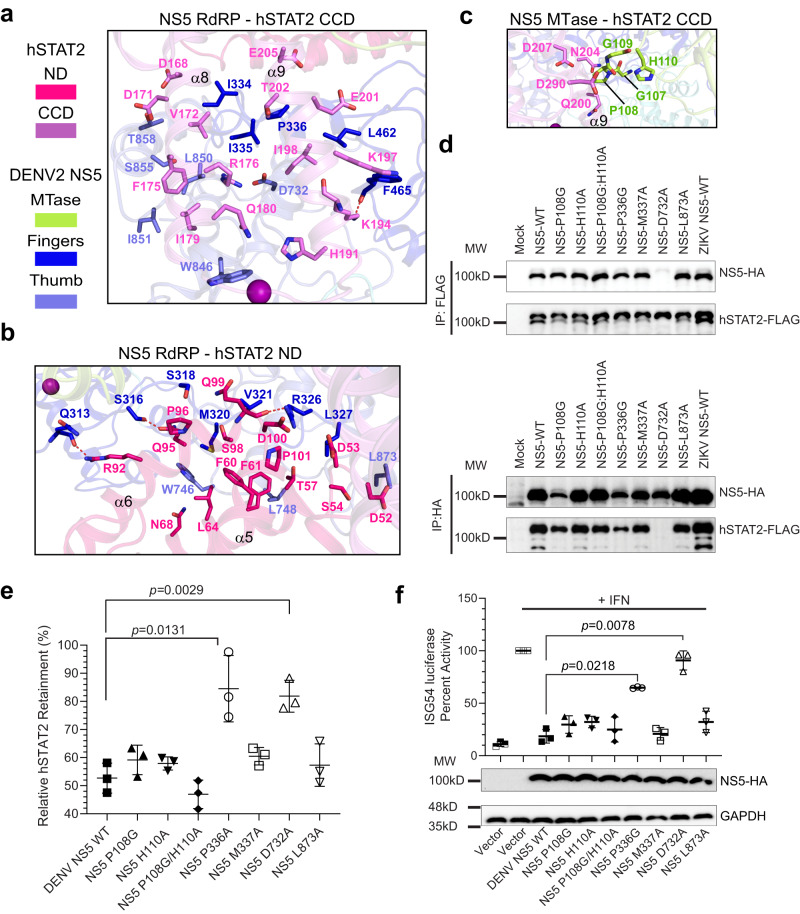
Molecular basis of the DENV2 NS5–hSTAT2 interaction. **a**–**c** Close-up view showing NS5 RdRP–hSTAT2 CCD interaction (**a**), NS5 RdRP–hSTAT2 ND interaction (**b**), and NS5 MTase–hSTAT2 CCD interaction (**c**). Hydrogen bonds are shown as dash lines. **d** Co-IP analysis showing the interaction between wild-type (WT) or mutant NS5 and hSTAT2. Immunoblot analysis of the IP was performed using antibodies against FLAG (top) and HA (bottom). **e** Relative protein retainment of hSTAT2 co-transfected without or with 800 ng of DENV2 NS5 plasmid, analyzed using the result of immunoblot analysis of 293 T cells transfected with indicated plasmids encoding WT or mutant NS5-HAs at increasing amounts. **f** ISG54 reporter assay performed in triplicate in 293 T cells transfected with the plasmids encoding ISG54 promoter firefly luciferase reporter, Renilla luciferase (for normalization), and DENV2 NS5-HA (wild type or mutant). Data are mean ± s.d. (*n* = 3 independent transfections). Statistical analysis used two-tailed Student’s *t*-test.

Detailed inspection of the interaction between DENV2 NS5 and hSTAT2 reveals three discrete binding interfaces: NS5 RdRP−hSTAT2 CCD, NS5 MTase−hSTAT2 CCD, and NS5 RdRP−hSTAT2 ND (Fig. 2a–c). At the NS5 RdRP−hSTAT2 CCD interface, the protruding ends of the first two helices of CCD, α8 and α9, pack against the Thumb and Fingers of the RdRP via van der Waals contacts, involving residues D168, D171, V172, F175, R176, I179, Q180, H191, K197, L198, E201, T202 and E205 of the CCD, residues I334, I335, P336, L462 and F465 of the Fingers, and residues D732, W846, L850, I851, S855 and T858 of the Thumb (Fig. 2a). In addition, residues R176 of the CCD forms an electrostatic contact with residue D732 of the Thumb, and residue K194 of the CCD is hydrogen-bonded to residue F465 of the Fingers (Fig. 2a). At the NS5 RdRP−hSTAT2 ND interface, the α5- and α6-helices of the ND interact with the Fingers of the RdRP via van der Waals contacts and/or hydrogen-bonding interactions, involving residues D53, R92, Q95, P96, S98, Q99 and D100 of the ND and residues Q313, S316, S318, M320, V321, R326, L327 of the Fingers (Fig. 2b). Furthermore, residues D52, S54, T57, F60, F61, L64 and N68 of the ND interact with residues W746, L748 and L873 of the Thumb via van der Waals contacts (Fig. 2b). In addition, a moderate intermolecular interaction was observed at the NS5 MTase−hSTAT2 CCD interface, mediated by van der Waals contacts involving residues Q200, N204, D207 and D290 of the CCD and residues G107-H110 of the MTase (Fig. 2c).

Together, the intermolecular interactions at the NS5 RdRP−hSTAT2 ND, NS5 RdRP−hSTAT2 CCD, and NS5 MTase−hSTAT2 CCD interfaces result in buried surface area of 765 Å^2^, 833 Å^2^, and 92 Å^2^, respectively. It is worth noting that the N-terminal tail of DENV2 NS5 that was proposed for binding to UBR4 is positioned well beyond the hSTAT2-binding sites (Fig. 1b)^9^.

### Cellular analysis of the DENV2 NS5-mediated hSTAT2 binding and inhibition

To provide the molecular basis for the DENV2 NS5−hSTAT2 interaction at a cellular level, we selected a number of residues around the hSTAT2-interacting sites of DENV2 NS5 (P108G, H110A, P108G/H110A, P336G, P108G/H110A/P336G, M337A, D732A, L873A) for mutagenesis and co-transfected each with wild-type (WT) hSTAT2 into 293 T cells. Total protein was extracted from each group 48 hours post-transfection. Western blot analysis of whole cell extraction (WCE) reveals similar expression levels of WT and mutant NS5 proteins (Supplementary Fig. 4a), indicating minimal impact of those NS5 mutations on protein expression. Subsequent co-immunoprecipitation (Co-IP) analysis revealed that, in comparison with WT DENV2 NS5, the NS5 D732A mutation nearly abolished the hSTAT2 binding (Fig. 2d and Supplementary Fig. 4b, c), supporting a critical role of the NS5 D732A−hSTAT2 R176 ionic pair in complex formation (Fig. 2a). On the other hand, the rest of NS5 mutations led to either a modest reduction (for P336G) or no appreciable change of hSTAT2 binding (Fig. 2d and Supplementary Fig. 4b, c), in line with the observation that the DENV2 NS5−hSTAT2 association is dominated by collective van der Waals contacts (Fig. 2a–c). Nevertheless, the combined P108G/H110A/P336G triple mutation (Mut^TM^) led to more pronounced binding defect than the P336G mutation or P108G/H110A mutation both in vitro and in cells (Supplementary Fig. 4d–h), supporting the notion on the collective contribution of these sites to the DENV2 NS5−hSTAT2 interaction.

To interrogate how the DENV2 NS5−hSTAT2 interaction impacts hSTAT2 protein stability in cells, we performed the degradation analysis of hSTAT2 protein in the presence of co-transfected DENV2 NS5. At 48 h. post transfection, the protein level of hSTAT2 underwent a gradual decrease with increasing level of WT NS5 (Supplementary Fig. 5a), consistent with what was previously observed^7,14^. In contrast, the degradation of hSTAT2 was significantly attenuated in cells co-transfected with hSTAT2 and NS5 D732A, P336G or mut^TM^ mutants (Fig. 2e and Supplementary Fig. 5a, b), in line with the fact that these mutations led to impaired DENV2 NS5−hSTAT2 interaction (Fig. 2d and Supplementary Fig. 4). Consistent with the protein degradation assay, our ISG54 luciferase reporter assay, which links STAT2-mediated transcriptional regulation to a luciferase activity^9,31^, shows that relative to WT NS5, the D732A, P336G, and Mut^TM^ mutants all led to a significant increase in luciferase activity after IFN treatment, indicating an impaired suppression of IFN responses (Fig. 2f and Supplementary Fig. 5c). These observations lend a strong support to the notion that the direct interaction between DENV2 NS5 and hSTAT2 promotes protein degradation of the latter^7,14^.

### A conformational selection mechanism underlying the DENV2/ZIKV NS5−hSTAT2 interaction

**Fig. 3:**
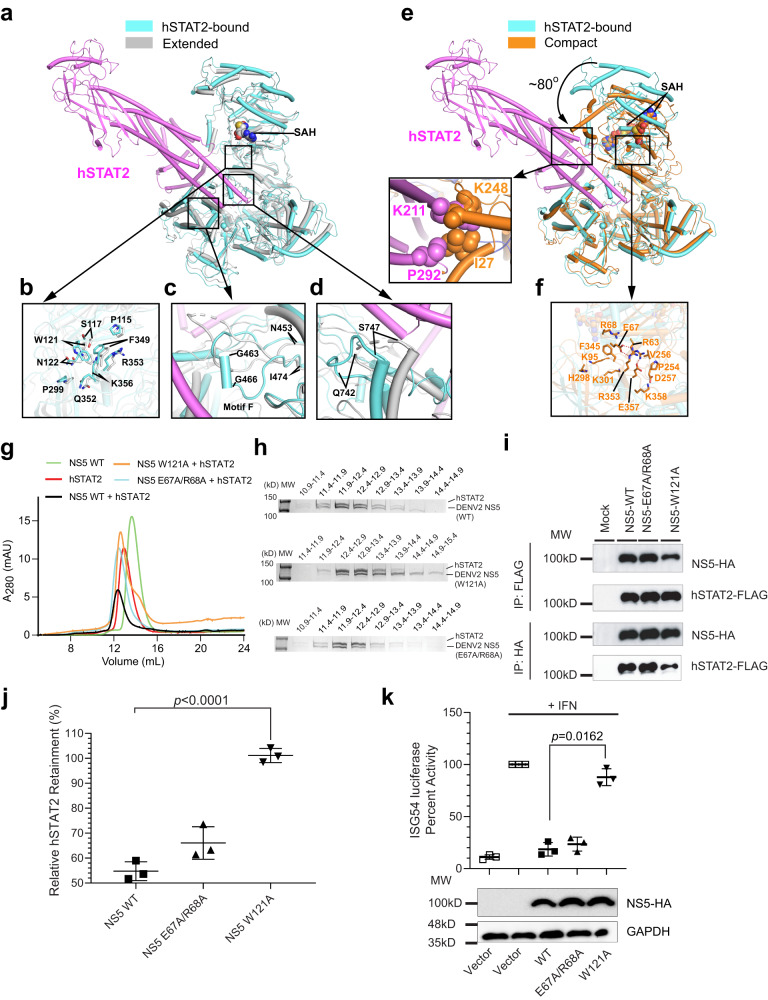
Conformational selection of DENV2 NS5 in the NS5–hSTAT2 interaction. **a** Structural superposition between hSTAT2-bound DENV2 NS5 and the extended conformation of its free form (PDB 6KR2). **b** Close-up view of the residues involved in the MTase–RdRP domain interaction in the extended conformation of free DENV2 NS5 and hSTAT2-bound DENV2 NS5. **c**, **d** Close-up view of the regions that show structural difference between the extended form of free and hSTAT2-bound DENV2 NS5. **e** Structural superposition between hSTAT2-bound DENV2 NS5 and the compact conformation of its free form (PDB 6KR3), with steric clash between hSTAT2 CCD and the compact form of free DENV2 NS5 shown in expanded view. **f** Close-up view of the residues involved in the MTase–RdRP domain interaction in the compact conformation of free DENV2 NS5. **g** Size-exclusion chromatography analysis of the interaction between hSTAT2 and DENV2 NS5, WT or mutant, using the elution profiles of apo hSTAT2 and DENV2 NS5 proteins as control. **h** SDS-PAGE analysis of the chromatography fractions derived from (**g**). **i** Co-IP analysis showing the interaction between WT or mutant NS5 and hSTAT2. Immunoblot analysis of the IP was performed using antibodies against FLAG (top) and (HA). **j** Relative protein retainment of hSTAT2 co-transfected without or with 800 ng of DENV2 NS5 plasmid, analyzed using the result of immunoblot analysis of 293 T cells transfected with indicated plasmids encoding WT or mutant NS5-HAs at increasing amounts. **k** ISG54 reporter assay performed in triplicate in 293 T cells transfected with the plasmids encoding ISG54 promoter firefly luciferase reporter, Renilla luciferase (for normalization), and DENV2 NS5-HA (wild type or mutant). Data are mean ± s.d. (*n* = 3 independent transfections). Statistical analysis used two-tailed Student’s *t*-test.

Considering that DENV2 NS5 in apo form exhibits at least two alternative conformations in solution^22,23^, we next asked how the hSTAT2 binding interplays with the conformational dynamics of DENV2 NS5. To address this, we superposed the structure of hSTAT2-bound DENV2 NS5 with the extended (PDB 6KR2) and compact (PDB 6KR3) forms of DENV2 NS5, respectively (Fig. 3a–f). The hSTAT2-bound form of DENV2 NS5 aligns well with the extended form of DENV2 NS5, with root-mean-square deviation (RMSD) of 2.6 Å over 854 aligned Cα atoms (Fig. 3a). Of note, the MTase−RdRP interfaces of DENV2 NS5 is maintained by the same set of residues, involving residues P115, S117, W121 and N122 from the MTase domain and residues P299, F349, Q352, R353 and K356 of the RdRP domain (Fig. 3b). The major structural difference between the hSTAT2-bound DENV2 NS5 and the extended form of apo DENV2 NS5 lies in the Fingers and Thumb of the RdRP domain. For instance, a substrate-engaging motif (motif F: residues 453-474) is extended toward the MTase−RdRP interface in the extended form of apo DENV2 NS5 but becomes retracted to interact with the CCD of hSTAT2 via a short helix (residues G463-G466) in the DENV2 NS5−hSTAT2 complex (Fig. 3c). In addition, residues Q742-S747 undergo a disorder-to-order transition in response to their binding to hSTAT2 (Fig. 3d). On the other hand, a large conformational difference is observed between hSTAT2-bound DENV2 NS5 and the compact form of apo DENV2 NS5, with an RMSD of 11.6 Å over 840 aligned Cα atoms (Fig. 3e). In fact, detailed comparison between the two structures reveals a potential steric clash between free DENV2 NS5 in the compact conformation and hSTAT2 (Fig. 3f). These observations suggest that the extended conformation of DENV2 NS5 was selected out of the conformational population for a specific interaction with hSTAT2.

As previously observed^22,23^, the two alternative conformations of DENV2 NS5 involve distinct sets of residues at the MTase−RdRP interface: the extended conformation resorts to residues P115-N122 of the MTase domain to interact with the RdRP; in contrast, the compact conformation enlists residues R63, E67, R68, K95, P254, V256 and D257 of the MTase domain for the RdRP interaction, resulting in a larger buried surface area (~848 A^2^ for the compact form vs ~587 A^2^ for the extended form) (Fig. 3b, f). Sequence analysis of flavivirus NS5 reveals that the residues for both interfaces are highly conserved within the flavivirus family (Supplementary Fig. 6)^6^, suggesting that both conformations may exist for many other, if not all, flavivirus NS5s. To test how the two alternative conformations of NS5 contributes to the hSTAT2 recognition, we generated interface mutations that disrupt either the compact conformation (E67A/R68A) or the extended conformation (W121A) of DENV2 NS5 or ZIKV NS5 and evaluated their impact on the DENV2/ZIKV NS5−hSTAT2 interaction via size-exclusion chromatography (Fig. 3g, h). At 350 mM NaCl concentration, we observed that WT ZIKV NS5 co-elutes with hSTAT2, indicative of formation of the ZIKV NS5−hSTAT2 complex (Supplementary Fig. 7a). On the other hand, introducing the NS5 W121A, but not the E66A/R67A mutation, led to disrupted co-migration between ZIKV NS5 and hSTAT2, supporting the notion that the extended conformation, but not the compact conformation of NS5, is essential for the complex formation between ZIKV NS5 and hSTAT2. For the DENV2 NS5−hSTAT2 complex, neither DENV2 E67A/R68A nor DENV2 W121A mutation led to appreciable complex disruption under the same salt concentration (Supplementary Fig. 7b), reflecting a higher stability of the DENV2 NS5−hSTAT2 complex than that of the ZIKV NS5−hSTAT2 complex. Nevertheless, when increasing the salt concentration to 1 M NaCl, it is evident that the complex of DENV2 NS5-hSTAT2 is substantially disrupted by the NS5 W121A mutation, but not by the NS5 E67A/R68A mutation (Fig. 3g, h). These observations support that both DENV2 NS5 and ZIKV NS5 bind to hSTAT2 in a conformational selection mechanism.

To evaluate the impact of the conformational states of DENV2 NS5 in hSTAT2 inhibition, we further co-transfected 293 T cells with DENV2 NS5, either WT or the interface mutants, and hSTAT2. Our co-IP analysis shows that hSTAT2 binds strongly to both WT and E66A/R67A DENV2 NS5, whereas the binding is greatly impaired by the NS5 W121A mutation (Fig. 3i). Consistently, our cellular protein degradation analysis indicates that the protein level of hSTAT2 is significantly reduced with increasing concentrations of WT or E66A/R67A DENV2 NS5, but not W121A DENV2 NS5 (Fig. 3j and Supplementary Fig. 7c). In line with these observations, our ISG54 luciferase reporter assay reveals that the W121A mutant of DENV2 NS5 induced a significant higher luciferase activity than WT NS5 (Fig. 3k), indicating a loss of functionality of the W121A mutant in suppressing IFN response. These data further support that a conformational selection mechanism underlies flavivirus NS5-mediated hSTAT2 degradation.

It is worth noting that the structures of DENV3 NS5 replication complexes have recently been reported^25^. Structural alignment of the promoter complex of DENV3 NS5 (PDB 8GZP), in which DENV3 NS5 binds to the stem-loop A (SLA) of the viral genome, with the compact conformation of DENV2 NS5 (PDB 6KR3) gives an RMSD of 1.5 Å over 787 aligned Cα atoms (Supplementary Fig. 8a), indicative of a highly similar compact conformation. In contrast, a large structural difference was observed between the promoter complex of DENV3 NS5 and the DENV2 NS5−hSTAT2 complex, with an RMSD of 12.3 Å over 772 aligned Cα atoms (Supplementary Fig. 8b). Notably, the presence of DENV3 NS3 in the RNA elongation complex transits the DENV3 NS5 into an open conformation with much reduced inter-domain contact (buried surface area of ~448 A^2^), distinct from both the compact and extended conformations (Supplementary Fig. 8c–e). These observations highlight that DENV NS5 resorts to distinct conformations for hSTAT2 binding and RNA replication.

### Structural comparison of the flavivirus NS5−hSTAT2 interaction

**Fig. 4:**
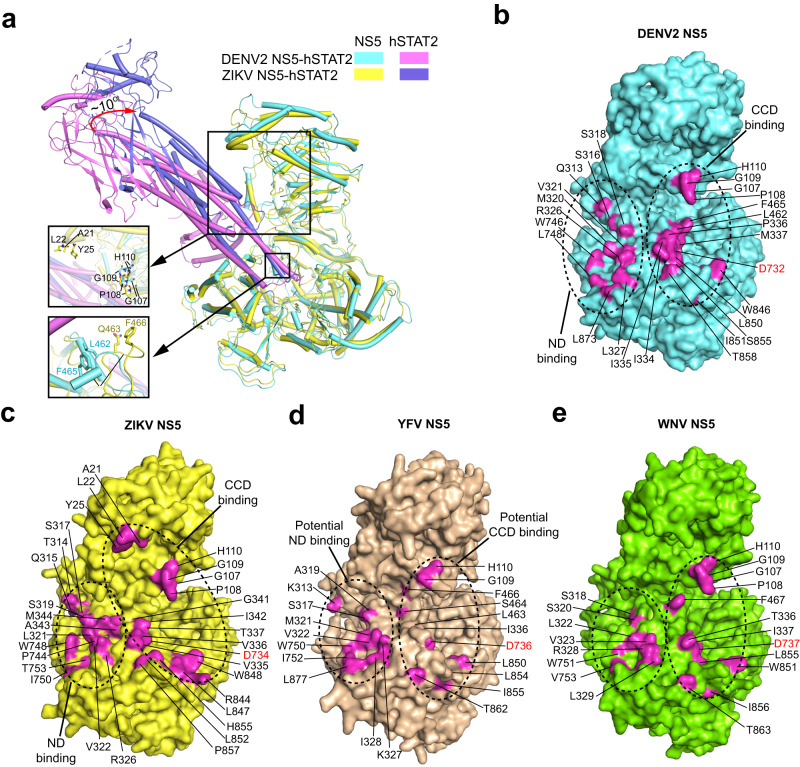
Molecular basis for the species-specific flavivirus NS5–hSTAT2 binding. **a** Structural superposition between hSTAT2-bound DENV2 NS5 and ZIKV NS5 (PDB 6WCZ), with two structurally divergent regions shown in expanded views. **b** Surface mapping of the hSTAT2-interaction sites (magenta) in DENV2 NS5. The hSTAT2 ND- and CCD-binding sites are circled in dotted lines. **c** Surface view of the hSTAT2-interaction sites (magenta) in ZIKV NS5 (PDB 6WCZ). **d** Surface view of the potential hSTAT2-interaction sites (magenta) of YFV NS5 (PDB 6QSN), identified by sequence and structural comparison with DENV2 NS5. **e** Structural model of full-length WNV NS5, with residues bearing sequence and structural similarity to the hSTAT2-binding sites in DENV2 NS5 colored in magenta.

To understand how flavivirus NS5 has evolved into a protein-interaction platform for suppression of IFN signaling, we performed structural comparison between DENV2 NS5−hSTAT2 and ZIKV NS5−hSTAT2 complexes. Alignment of DENV2 NS5−hSTAT2 complex with the cryo-EM structure of ZIKV NS5−hSTAT2 complex (PDB 6WCZ), in which hSTAT2 ND is disordered, reveals high similarity, with RMSD of 3.6 Å over 1114 aligned Cα atoms (Fig. 4a). Likewise, superposition of the DENV2 NS5−hSTAT2 complex with the crystal structure of ZIKV RdRP−hSTAT2 complex (PDB 6UX2), in which hSTAT2 ND is partially traced, also reveals similar conformations and intermolecular contacts, with RMSD of 3.1 Å over 976 aligned Cα atoms (Supplementary Fig. 9a). Of note, in both DENV2 NS5−hSTAT2 and ZIKV NS5−hSTAT2 complexes, the NS5 protein adopts an extended conformation, with the MTase−RdRP interdomain cleft embracing the protruding end of the hSTAT2 CCD (Fig. 4a). Accordingly, DENV2 NS5 and ZIKV NS5 similarly involve three distinct interfaces for hSTAT2 interaction: NS5 RdRP−hSTAT2 CCD, NS5 MTase−hSTAT2 CCD and NS5 RdRP−hSTAT2 ND (Figs. 2a–c, 4a–c and Supplementary Fig. 9a). Nevertheless, notable divergence in hSTAT2 interaction was observed for DENV2 NS5 and ZIKV NS5. For instance, motif-F residues L462 and F465 of DENV2 NS5 engage in an interaction with the hSTAT2 CCD, whereas the corresponding sites (Q463 and F466) of ZIKV NS5 failed to contact hSTAT2 due to a conformational difference of this region (Fig. 4a). Furthermore, the MTase domain of ZIKV NS5 interacts with hSTAT2 CCD via both the GPGH (G107-P108-G109-H110) loop and an N-terminal helix (residues A21, L22 and Y25) (Fig. 4c), whereas the DENV2 MTase only resorts to the GPGH loop for hSTAT2 interaction. In addition, residue variations between DENV2 NS5 and ZIKV NS5 were observed for the NS5 RdRP−hSTAT2 ND interface (Fig. 4b, c). It is worth noting that, in contrast to the cryo-EM structure of DENV2 NS5−hSTAT2 where the hSTAT2 ND is fully defined, the crystal structure of ZIKV RdRP−hSTAT2 (PDB 6UX2) contains partially disordered hSTAT2 ND (Supplementary Fig. 9a). Whether such a difference arises from the experimental conditions or intrinsic conformational properties of DENV2 NS5 and ZIKV NS5 awaits further investigation. Nevertheless, the residue variations for the hSTAT2-interacting sites between DENV2 NS5 and ZIKV NS5 provide an explanation for their differential binding activities toward hSTAT2 (Supplementary Fig. 7a, b).

To understand how hSTAT2-binding affinity varies among flavivirus NS5s, we further analyzed the structure of full-length YFV NS5 (PDB 6QSN) that reportedly binds to hSTAT2 upon IFN activation^9^. In addition, we generated a structural model for full-length WNV NS5, which reportedly suppresses IFN signaling via targeting prolidase, rather than hSTAT2 as shown in our size-exclusion chromatography analysis (Supplementary Fig. 9b)^13^, using the SWISS-model server (https://swissmodel.expasy.org/), with the extended conformation of DENV2 NS5 (PDB 6KR2) as template. Compared with DENV2 NS5 and ZIKV NS5, both YFV NS5 and WNV NS5 show a high sequence and structural similarity on the hSTAT2-binding sites, including residues that correspond to DENV2 NS5 G109-H110, V321, L327, D732, W746, L850, I851, and T858, etc. (Fig. 4b–e and Supplementary Fig. 6). These observations suggest that the hSTAT2-binding sites constitute a conserved protein-interaction platform of flavivirus NS5, whereas their divergent target specificity toward the IFN signaling pathway may arise from subtle variations in sequence, structure and/or dynamics of this platform. The molecular basis underpinning the varied hSTAT2-binding affinities of YFV NS5 and WNV NS5 await further investigation.

## Discussion

Flavivirus NS5 is an attractive therapeutic target owing to its role in both genome replication of virus and antagonism of host IFN signaling^5,6^. Yet, how the family of flavivirus NS5 proteins has evolved into a protein interaction platform to target diverse IFN players remains elusive. This study, through structural, biochemical and cellular characterization of the interaction between DENV2 NS5 and hSTAT2 in the evolutionary context, identified a highly conserved protein-interaction mechanism for flavivirus NS5, unraveling an interplay between its conformational dynamics and species-specific interaction with STAT2 or other IFN-associated protein factors.

First, our study demonstrated that both DENV2 NS5 and ZIKV NS5 proteins engage with hSTAT2 through one of their preconfigured conformations. Previous biochemical and structural investigations have established that flavivirus NS5 proteins can adopt two distinct domain conformations: a compact form and an extended form^17–21,23,24^. Although the precise functional roles of these conformational states remain elusive, their existence offers the NS5 protein versatility in interacting with various host proteins. In line with this notion, recent studies revealed that DENV NS5-mediated RNA replication involves multiple conformational states^25^, and that disruption of either the compact or the extended conformation of DENV NS5 compromised viral proliferation^23^. Building upon these findings, the cryo-EM structure of the DENV2 NS5−hSTAT2 complex presented in this study unveils that both DENV2 NS5 and ZIKV NS5 proteins rely on their extended forms to interact with hSTAT2. Notably, disruption of the extended conformation, rather than the compact conformation, led to the impairment of NS5−hSTAT2 interaction, supporting a conformational selection mechanism governing the interaction of flavivirus NS5 with hSTAT2 or other potential host targets. The consequences of this functional interplay are twofold: On one hand, the NS5−hSTAT2 interaction may facilitate the proteasome-mediated degradation of hSTAT2, thereby influencing host immune response; on the other hand, it also leads to the inhibition of NS5-mediated RNA synthesis during the early stages of infection, which underscores the delicate balance between viral replication and immune evasion.

Second, this study provides insight into how flavivirus NS5s, the most conserved viral protein within the flavivirus family, have evolved with a broad spectrum of target specificities in IFN suppression. Previous studies have revealed that DENV NS5, ZIKV NS5, and YFV NS5 interact with hSTAT2 to promote its proteasome-mediated degradation (in the case of DENV and ZIKV) or prevent its binding to IFN-stimulated responsive element (ISRE) promoter elements (in the case of YFV)^7,9,11,14^. In contrast, whereas SPOV NS5, the closer relative of ZIKV NS5, inhibits JAK-STAT signaling, it does not interact with hSTAT2^9^. In addition, WNV NS5 was found to target the host protein prolidase to inhibite surface expression of the IFNAR1 receptor, which is crucial for the IFN signaling pathway^13^. Here, our structure-functional analysis reveals that DENV2 NS5 engages in multivalent interactions with hSTAT2, involving surface regions that are similar to those for the ZIKV NS5-hSTAT2 interaction. These interactions are dominated by surface complementarity and collective van der Waals contacts, opening room for tuning protein interaction affinity and specificity through subtle amino acid variations. This observation suggests that all flavivirus NS5 proteins likely rely on the same surface regions for host interaction but attain distinct substrate specificity via fine tuning of a subset of amino acids. Consistent with this notion, members of flavivirus NS5 family show high sequence and structural conservation in these regions, despite with distinct substrate specificities. These observations raise an intriguing possibility that flaviviruses may employ a common platform for suppressing the IFN response via diverse protein interactions.

Finally, this study also reinforces the important role of hSTAT2 CCD in protein interaction. Structures for several hSTAT2-mediated protein complexes have recently been reported, including the STAT2−IRF9^34^, STAT2−DDB1−RCMV E27^35^, STAT2−DENV2 NS5 and STAT2−ZIKV NS5^31^ complexes. Common to all these complexes is that hSTAT2 employs the same protruding end of the CCD for the intermolecular interactions (Supplementary Fig. 10). These observations suggest that that hSTAT2 CCD serves as an interaction hotspot for STAT2, highlighting a multifaceted role of this domain in STAT2 function.

Overall, this study sheds light on the intricate interplay between flavivrus NS5 proteins and hSTAT2, elucidating the role of conformational dynamics in flavivirus-host interactions. These findings open an avenue for the development of therapeutic strategies to combat flavivirus infections.

## Methods

### Cloning, protein expression and purification

The cDNA fragments encoding full-length human STAT2 (hSTAT2), DENV2 NS5 and WNV NS5 were each cloned into a modified pRSF vector, in which the hSTAT2 or DENV2 NS5 gene was preceded by an N-terminal His_6_-SUMO tag and ULP1 (ubiquitin-like protease) cleavage site. The plasmids were transformed into BL21 RIL (DE3) cell strain (Agilent technologies) for protein expression. The transformed cells were first grown at 37 °C until cells attained an OD_600_ of 0.8. The temperature was then lowered to 16 °C, after which the cells were induced by addition of 0.1 mM isopropyl β-D-galactoside and continued to grow overnight. For His_6_-SUMO-tagged hSTAT2, the fusion protein was purified using a Ni-NTA affinity column, followed by removal of the His_6_-SUMO tag with ULP1 protease, ion exchange chromatography on a Heparin HP column (GE Healthcare), and size-exclusion chromatography on a HiLoad 16/600 Superdex 200 pg column (GE Healthcare) pre equilibrated with 25 mM Hepes (pH 7.5), 0.25 M NaCl, 5% Glycerol and 5 mM DTT. Purification of DENV2 NS5, ZIKV NS5 and WNV NS5 was similar to that for hSTAT2, except that after Ni-NTA affinity chromatography and removal of the His_6_-SUMO tag, the NS5 protein was further purified by hydrophobic interaction chromatography using a Butyl-sepharose column (GE Healthcare), followed by size-exclusion chromatography on a HiLoad 16/600 Superdex 200 pg column (GE Healthcare) pre-equilibrated with 25 mM Hepes (pH 7.5), 500 mM NaCl, 5% Glycerol and 5 mM DTT. The purified proteins were confirmed by SDS-PAGE analysis, concentrated to ~10–20 mg/mL and stored at −80 °C for further use.

### Cryo-EM sample preparation and data acquisition

The protein sample of full-length hSTAT2 was mixed with full-length DENV2 NS5 in a 1:1 molar ratio, followed by size-exclusion chromatography on a Superdex 10/300 increase gl column (GE Healthcare) pre-equilibrated with buffer containing 25 mM Hepes (pH 7.5), 175 mM NaCl, and 5 mM DTT. The peak fraction was collected and concentrated to ~0.8 mg/mL before use.

For cryo-EM sample preparation, an aliquot of 2.5 μL of the above optimized hSTAT2−DENV NS5 complex at a concentration of approximately 0.5 mg/mL was applied to a Quantifoil holey carbon grid (Copper,1.2/1.3, 300 mesh), which was glow discharged for 1 min with a PELCO Easy Glow system. The grid was blotted and plunge-frozen in liquid ethane cooled by liquid nitrogen with a Vitrobot IV (Thermo Fisher) at 4 °C under 100% humidity. The frozen grids were stored in liquid nitrogen before use.

High-resolution cryo-EM data were collected on a Titan Krios electron microscope operating at 300 kV and equipped with a post-GIF Gatan K3 Summit (BioQuantum) direct electron detector via the Latitude software (Gatan, Inc) in the National Cryo-Electron Microscopy Facility of Fredrick National Laboratory for Cancer Research. Movies were recorded at a nominal magnification of ×81,000 with a pixel of 1.11 Å in counting mode. Each micrograph was recorded 40 frames at a dose rate of 12.5 *e*^*-*^/ Å ^2^/s and total expose time of 4 s, resulting a total dose rate of 50 *e*^*−*^/Å^2^. The norminal defocus range was set to −1.0 to −2.0 µm.

### Cryo-EM data processing

The cryo-EM data for the DENV2 NS5−hSTAT2 complex were processed using cryoSPARC (v3.3.1)^33^. The movies were motion-corrected and dose-weighted using the patch-based motion correction module. The contrast transfer function (CTF) of the resulting images, with a pixel size of 1.1 Å, were then subject to patch-based estimation. Automatic particle picking was performed using the TOPAZ method^36^. After training and particle picking with TOPAZ, 1,574,562 particles were extracted from 2909 images, with a down-scaled pixel size of 3.1 Å. After two rounds of clean-up by 2D classifications, those classes with identifiable secondary structures and/or defined features of a tertiary fold, comprised of 1,139,742 particles, were selected. Initial models were then generated by 3D ab initio reconstruction followed by one round of heterogenous refinement applying C1 symmetry. After another round of 3D classification, 235,139 particles were selected and re-extracted with a pixel size of 1.1 Å. Subsequently, a final round of 3D classification was performed, leading to a class (108,309 particles) with defined density for the N-terminal domain (ND) of hSTAT2 as well as other interaction domains of both proteins, and another class (92,065 particles) lack of density for the hSTAT2 ND. After another round of clean up by 2D classification, 99,938 and 86,928 particles were then refined respectively for the hSTAT2 ND-ordered and ND-disordered complexes using non-uniform refinement module within the CryoSPARC software package. The average resolution was given by the Fourier shell correlation criterion (FSC 0.143). CryoSPARC was used to determine local resolution and sharpen the maps.

### Model building

For model building of the DENV2 NS5–hSTAT2 complex, the reported structures of the DENV2 NS5 (PDB 6KR2)^1^ and hSTAT2 (PDB 6UX2)^6^ were used to fit into the cryo-EM map in Chimera X (v1.4)^7^. The initial structural model was then subject to iterative model building using Coot (v0.9.6)^8^ and real-space refinement using Phenix (v1.8.2)^9^. A model-map Fourier shell correlation was calculated using the criterion of 0.5.

### Analytical size-exclusion chromatography

For size-exclusion chromatography analysis of the complexes of DENV2 NS5, ZIKV NS5 or WNV NS5 with hSTAT2, the NS5 samples were mixed with hSTAT2 protein N-terminally fused to a Strep-tag via Gly-Ser dipeptide linker in a stoichiometric ratio. In total 0.5 mL of the complex sample at a concentration of ~0.2 mg/mL was loaded onto Superdex 200 increase 10/300 gl column (GE Healthcare) and eluted using buffer containing 20 mM HEPES (pH 7.5), 250 mM NaCl or otherwise indicated salt concentrations, 5% glycerol and 5 mM DTT. The peak fractions were analyzed using SDS-PAGE.

### Plasmids and transfections

To express the DENV2 NS5 protein with the authentic N-terminus in 293 T cells, we resorted to the N-ubiquitin pCAGGS vector and cloned DENV2 NS5, wild type or one of the mutants, with an HA tag fused to the carboxy -terminus. All constructs were confirmed by Sanger sequencing.

### Co-immunoprecipitation

To investigate the impact of different DENV2 NS5 residues required for the interaction between human STAT2 and DENV2 NS5 proteins, we used PEI to co-transfect 1 × 10^6^/well 293 T cells with 0.5 µg of each plasmid: pCAGGS encoding hSTAT2-FLAG^31^ and different DENV2 NS5-HA constructs. After 48 hours post-transfection (hpt), the cells were harvested and lysed in NP-40 buffer [0.25% NP-40, 50 mM Tris (pH 7.4), 150 mM NaCl, 5 mM EDTA, 10% glycerol, and 1 mM PMSF]. After being incubated on ice for 30 minutes, the cell samples were centrifuged at 15,000 rpm at 4 °C for 10 minutes. The whole-cell extract (WCE) was used for immunoprecipitation by rotating end-over-end at 4 °C for 1 hour with 1 µg of monoclonal anti-FLAG antibody (catalog no. F3165, Sigma) or 1 µg of monoclonal anti-HA antibody (catalog no. 66006, Proteintech), followed by another 1-hour end-over-end rotation with recombinant protein G Sepharose 4B beads (catalog no. 101243, Thermo Fisher), pre-blocked with 5% BSA. The beads were pelleted, washed three times with PBS, and the bound proteins were eluted by boiling in a heat block. The Co-IPs and WCE were analyzed by SDS-PAGE, followed by immunoblotting using primary antibodies including mouse anti-GAPDH (catalog no. 60004, Proteintech), anti-HA, and anti-FLAG, and subsequently goat anti-mouse IgG (H + L) HRP-linked secondary antibody (catalog no. 20-304, Genesee Scientific).

### hSTAT2 degradation assay

Five ×10^5^/well 293 T cells were transfected with increasing amounts (0 ng, 200 ng, 400 ng, 800 ng) of designated pCAGGS plasmids encoding HA-tagged NS5 variants, along with 300 ng each of pCAGGS plasmids encoding hSTAT2-FLAG. PUG-19 was used to balance the DNA amount and eliminate variations in DNA usage among different samples. After 48 h of transfection, the cells were harvested in 1× Loading buffer and subjected to SDS–PAGE analysis. Immunoblotting was performed using primary antibodies against anti-GAPDH (catalog no. 60004, Proteintech), anti-HA (catalog no. 66006, Proteintech), and anti-FLAG (catalog no. F3165, Sigma), followed by secondary IgG antibodies (H + L) HRP-linked (catalog no. 20-304, Genesee Scientific) either anti-mouse or goat anti-mouse. The degree of degradation was calculated by dividing the densitometry reading of the hSTAT2 protein in the presence of exogeneous NS5 protein by the densitometry reading of the hSTAT2 protein without exogeneous NS5 protein. The results were summarized from three independently performed experiments.

### Luciferase reporter assay

We followed a previously reported protocol^9,31^ for ISG54 reporter assays. 293 T cells were co-transfected with one of the NS5-HA plasmids, the IFN-inducible firefly luciferase reporter (ISG54 promoter), and a plasmid constitutively expressing the renilla luciferase protein. Twenty-four hours post-transfection, cells were treated with 1000 U of universal IFN(PBL) and analyzed for reporter activity 12 h post-treatment. Average firefly luciferase values were firstly normalized to average renilla luciferase values. Empty vector treated with IFN was set to 100% reporter activity. Error bars represent mean ± s.d. Results are representative of three independent experiments with each one in triplicate.

### Statistics and reproducibility

For analysis of protein degradation assay and luciferase reporter assay, two-tailed Student’s *t*-test was used with a sample s. The *P* value of less than 0.05 was considered significant. All biochemical and cellular assays were performed two or more times with similar results, unless indicated otherwise.

### Reporting summary

Further information on research design is available in the Nature Portfolio Reporting Summary linked to this article.

### Supplementary information

Supplementary Information

Supplementary Data 1

Reporting Summary

Description of Additional Supplementary Files

## Data Availability

Coordinates and structure factors for the DENV2 NS5–hSTAT2 complex, with hSTAT2 ND ordered or disordered, have been deposited in the Protein Data Bank with accession codes 8T12 and 8T13, respectively. The cryo-EM density maps have been deposited to the EMDB and PDB under the accession numbers of EMD-40952 and EMD-40953, respectively. Source data underlying figures are provided in Supplementary Data 1, and raw SDS-PAGE and western blot images are provided in Supplementary Fig. 11.
