# Supplementary material for: A conformational selection mechanism of flavivirus NS5 for species-specific STAT2 inhibition
Source: Commun Biol. 2024 Jan 10;7:76. doi: 10.1038/s42003-024-05768-8 (PMC10776582; doi:10.1038/s42003-024-05768-8)
Supplement: Supplementary file 1 — Supplementary Information [file 42003_2024_5768_MOESM1_ESM.pdf]

Supplementary Information for

A conformational selection mechanism of flavivirus NS5 for species-specific STAT2 inhibition

Mahamaya Biswal[1,#], Wangyuan Yao[2,#], Jiuwei Lu[1], Jianbin Chen[1], Juliet Morrison[2], Rong Hai[2,*], Jikui Song[1,*]

[1]Department of Biochemistry, University of California, Riverside, California, USA

[2]Department of Microbiology and Plant Pathology, University of California, Riverside, California, USA

[#]These authors contributed equally to the work

*Correspondence should be addressed to J.S. (jikui.song@ucr.edu) or R.H. (ronghai@ucr.edu)

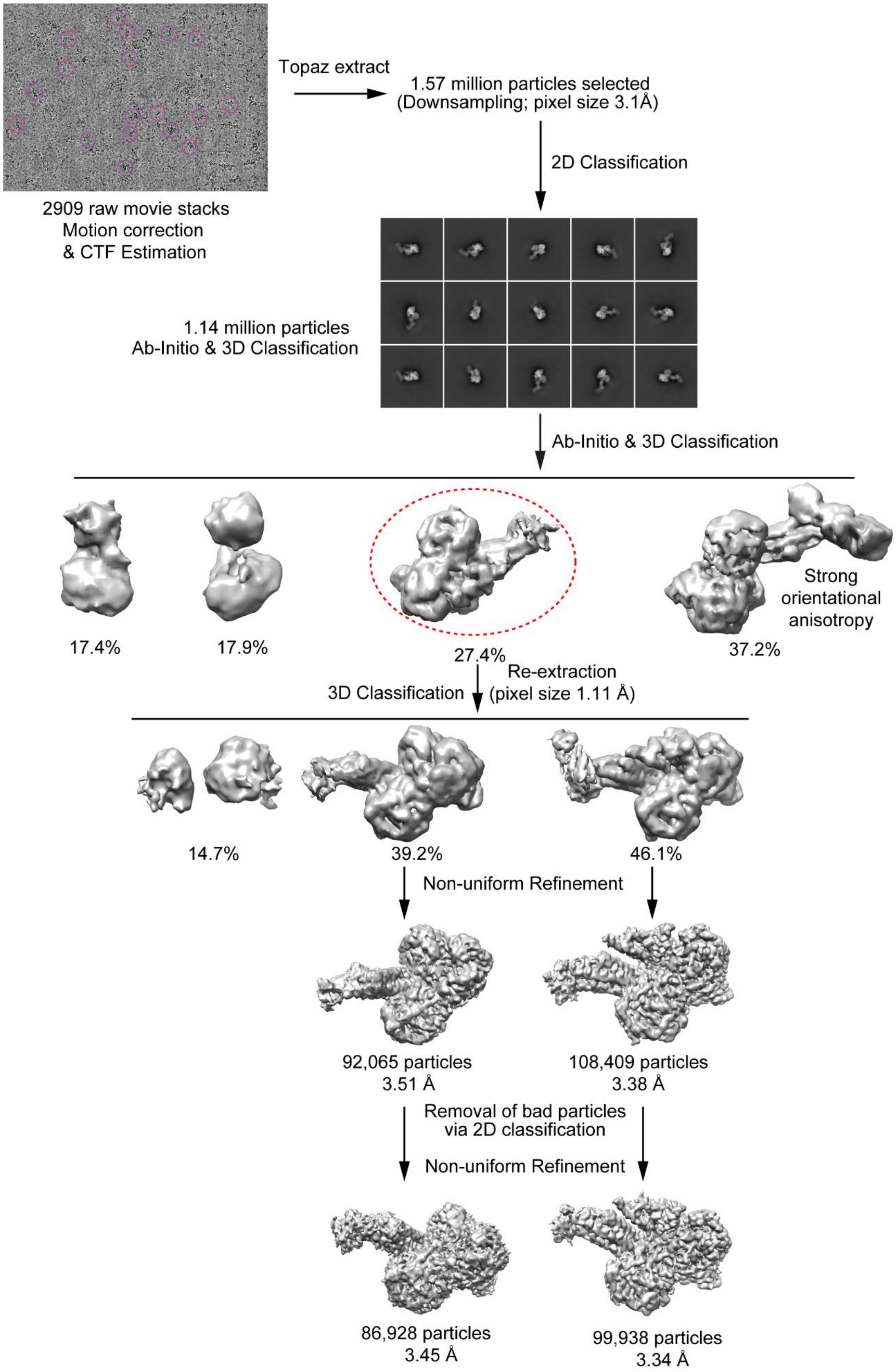

Topaz extract
1.57 million particles selected
(Downsampling; pixel size 3.1Å)
2909 raw movie stacks
Motion correction
& CTF Estimation
2D Classification
1.14 million particles
Ab-Initio & 3D Classification
Ab-Initio & 3D Classification
17.4%
17.9%
27.4%
Strong
orientational
anisotropy
37.2%
3D Classification
Re-extraction
(pixel size 1.11 Å)
14.7%
39.2%
46.1%
Non-uniform Refinement
92,065 particles
3.51 Å
108,409 particles
3.38 Å
Removal of bad particles
via 2D classification
Non-uniform Refinement
86,928 particles
3.45 Å
99,938 particles
3.34 Å

Supplementary Fig. 1. Data processing workflow for cryo-EM reconstruction of the DENV2 NS5–hSTAT2 complex. After initial 3D classification, the density map of the group subject to further classification is circled in dotted line.

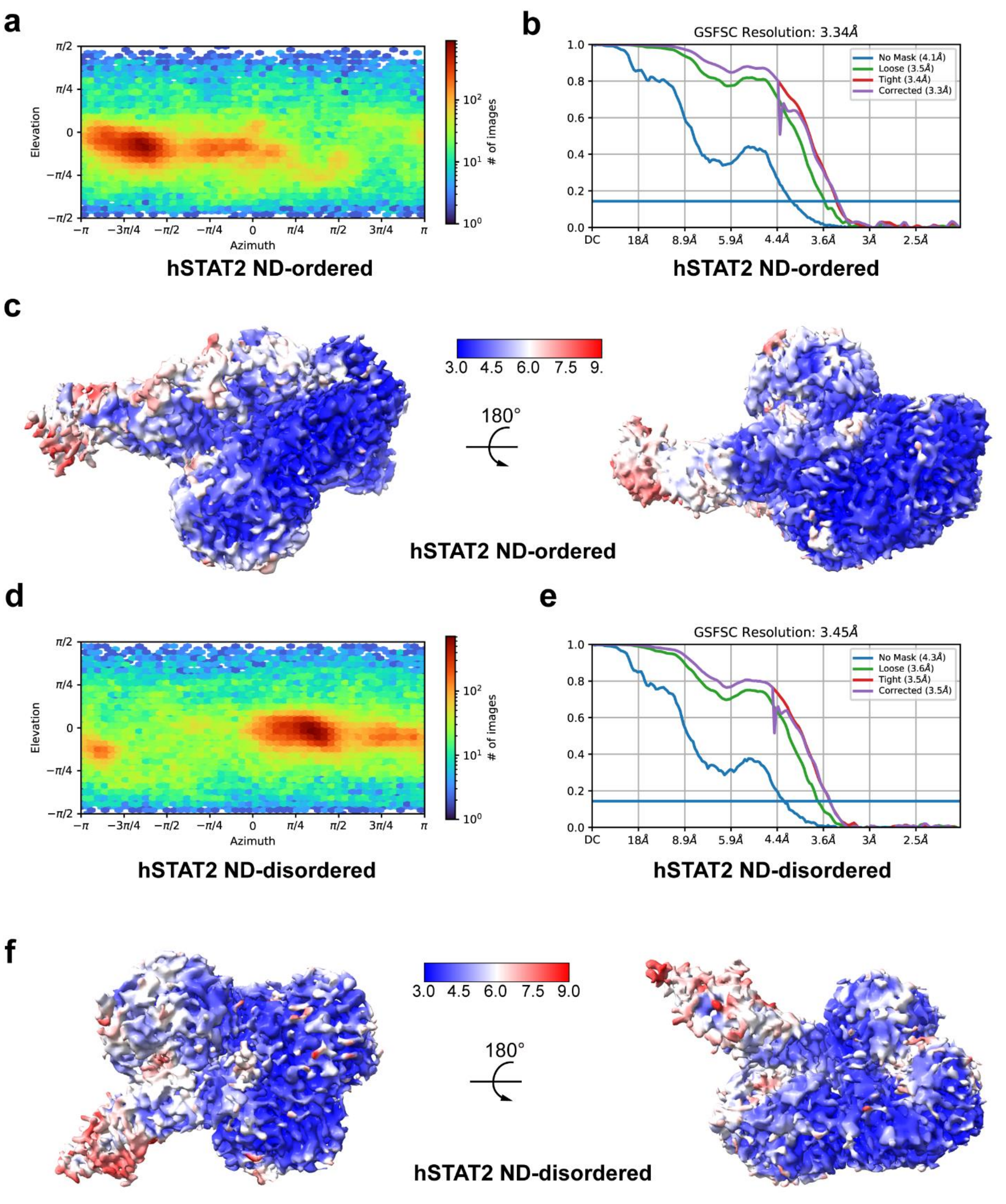

Supplementary Figure 2. Cryo-EM reconstruction of DENV2 NS5–hSTAT2 complex. **a**, Orientation distribution map of the DENV2–hSTAT2 complex with the hSTAT2 ND ordered. **b**, Fourier shell correlation (FSC) curve of the density map for the DENV2–hSTAT2 complex (hSTAT2 ND ordered) as a function of resolution using

cryoSPARC output. **c**, Local resolution map of the DENV2 NS5-hSTAT2 complex (hSTAT2 ND ordered). **d**, Orientation distribution map of the DENV2–hSTAT2 complex with the hSTAT2 ND disordered. **e**, Fourier shell correlation (FSC) curve of the density map for the DENV2–hSTAT2 complex (hSTAT2 ND disordered) as a function of resolution using cryoSPARC output. **f**, Local resolution map of the DENV2 NS5-hSTAT2 complex (hSTAT2 ND disordered).

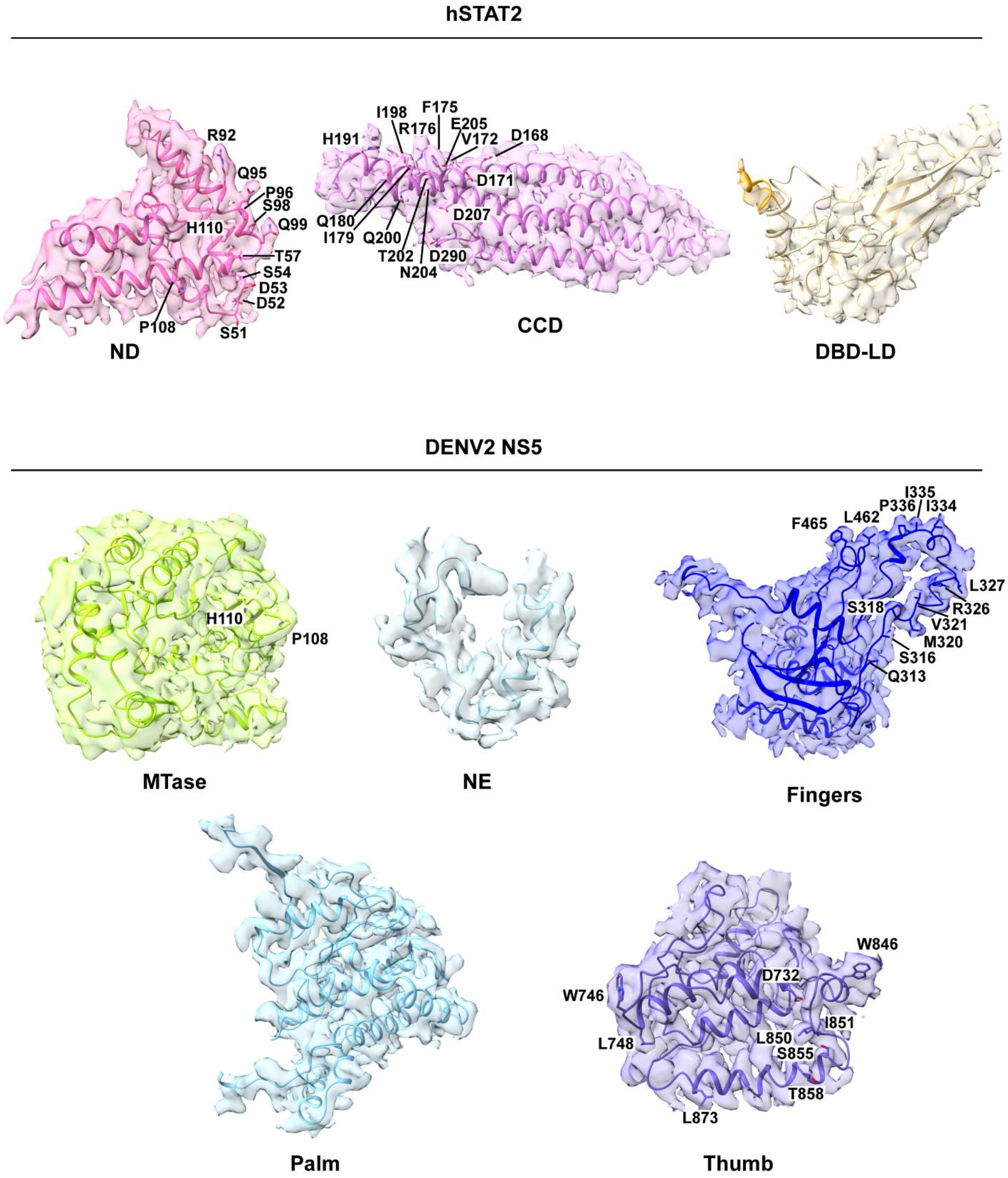

Supplementary Figure 3. Density maps of the individual domains within the DENV2 NS5–hSTAT2 complex. (top) Density maps of the individual domains of hSTAT2 within the DENV2 NS5–hSTAt2 complex. (bottom) Density maps of the individual domains of DENV2 NS5 within the DENV2 NS5–hSTAT2 complex.

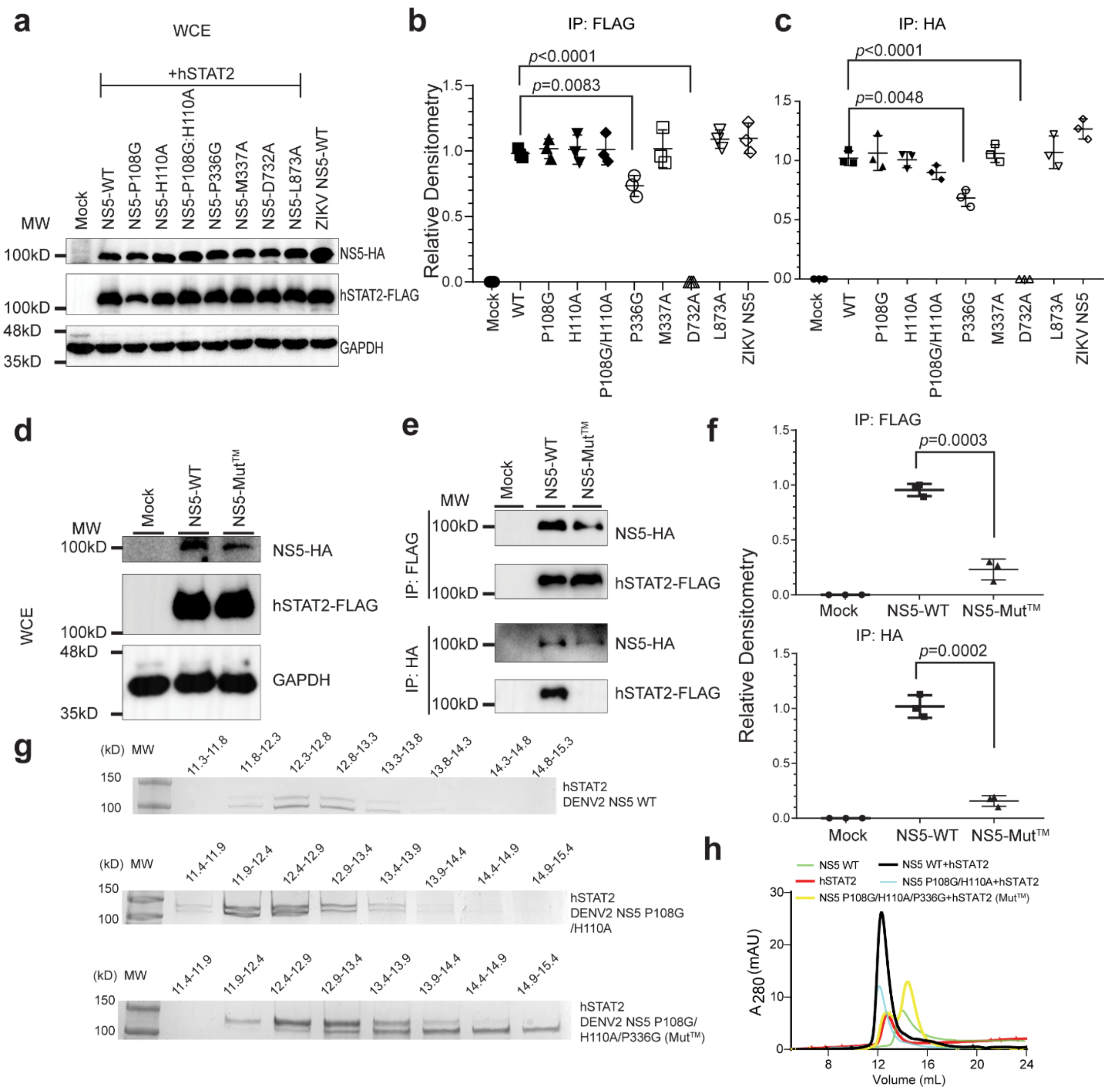

Supplementary Figure 4. Mutational analysis of the DENV2 NS5–hSTAT2 interaction. a, Immunoblot analysis of whole cell extract (WCE) of 293T cells, co-transfected with hSTAT2 and WT or mutant DENV2 NS5, was performed using antibodies against HA, FLAG and GAPDH. **b**, **c**, Relative densitometry of NS5-HA proteins co-transfected with hSTAT2-FLAG (b) or hSTAT2-FLAG protein co-transfected with WT or mutant NS5-HA (c), derived from immunoblot analysis in Fig. 2d. **d,** Immunoblot analysis of WCE of 293T cells, co-transfected with hSTAT2 and WT or P108G/H110A/P336G (mut^{TM}) mutant DENV2 NS5, was performed using antibodies against HA, FLAG and

GAPDH. **e**, Co-IP analysis showing the effects of Mut^{TM} NS5 mutation on the NS5–hSTAT2 interaction. Immunoblot analysis of the IP was performed using antibodies against FLAG (top) or HA (bottom). **f**, Relative densitometry of NS5-HA proteins co-transfected with hSTAT2-FLAG (top) or hSTAT2-FLAG protein co-transfected with WT or mutant NS5-HA (bottom), derived from immunoblot analysis in (d,e) respectively. **g, h,** SDS-PAGE analysis (g) of the size-exclusion chromatography fractions for WT, P108G/H110A, or mut^{TM} DENV2 NS5 protein (h), mixed with hSTAT2 in a stoichiometric ratio. The elution profiles for apo DENV2 NS5 and hSTAT2 were used as control. Data are mean ± s.d. (n=3 independent transfections). Statistical analysis used two-tailed Student's t-test.

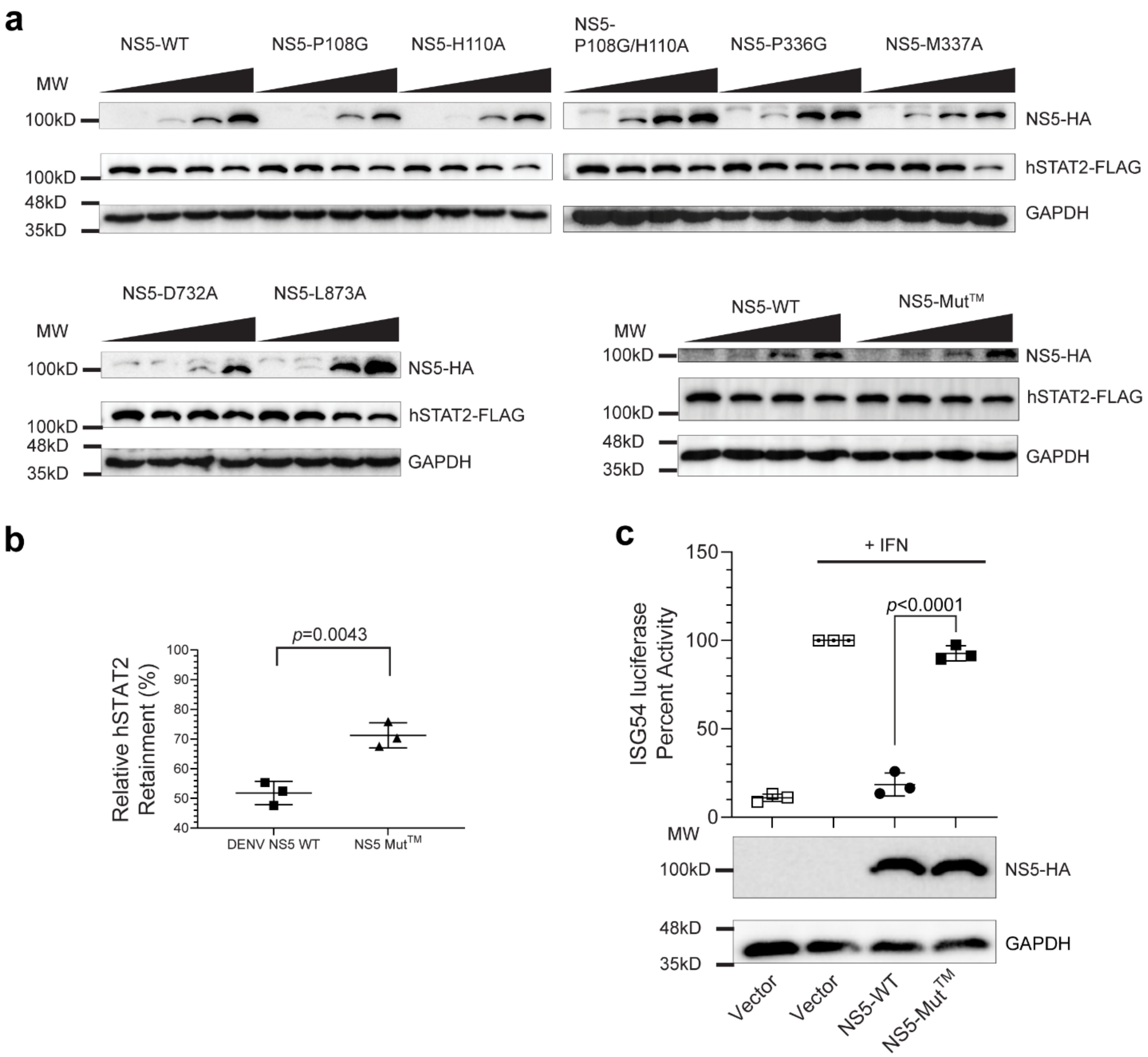

Supplementary Figure 5. Protein degradation assays for WT and mutant DENV2 DS5. a, Immunoblot analysis of 293T cells transfected with indicated plasmids encoding WT or mutant NS5-HAs at increasing amounts using antibodies against HA, FLAG, and GAPDH. Related to Fig. 2e. **b,** Relative protein retainment of hSTAT2 co-transfected without or with 800 ng of DENV2 NS5 plasmid, analyzed using the result of the corresponding panel in (a). **c**, ISG54 reporter assay performed in triplicate in 293T cells transfected with the plasmids encoding ISG54 promoter firefly luciferase reporter, Renilla luciferase (for normalization), DENV NS5-HA (WT or mut^{TM}). Data are mean ± s.d. (n=3 independent transfections). Statistical analysis used two-tailed Student's t-test.

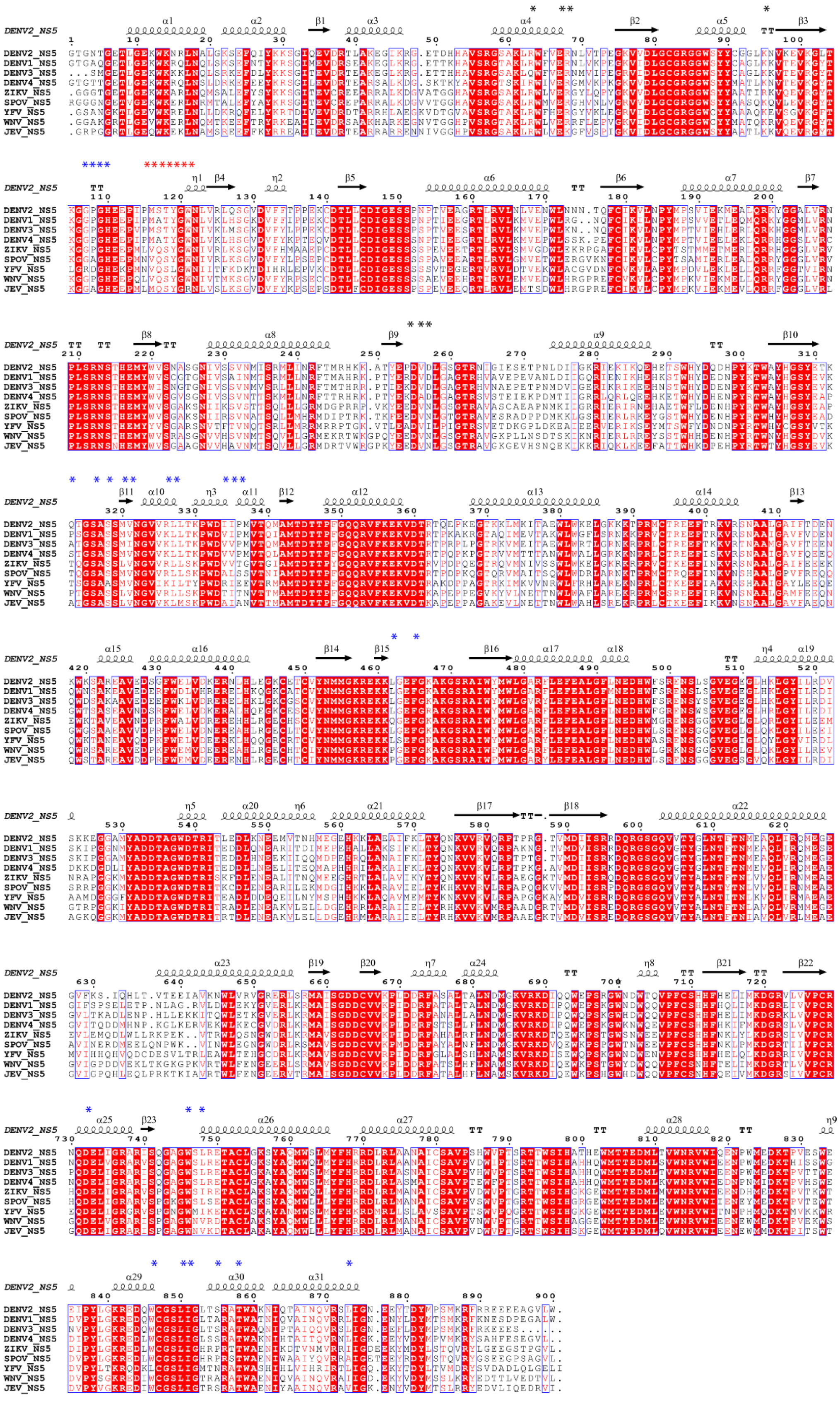

Supplementary Figure 6. Structure-based sequence analysis of the NS5 proteins from DENV2, DENV1, DENV3, DENV4, ZIKV, SPOV, YFV, WNV and JEV. The secondary structures of DENV2 NS5 are shown on top of the aligned sequences. Strictly conserved residues are colored white in red background. Similar residues are colored red. The hSTAT2-interacting residues of DENV2 NS5 are marked by blue asterisks on top. The residues of DENV2 NS5 mediating the MTase–RdRP domain interface for the compact and extended conformations are marked by black and red asterisks, respectively.

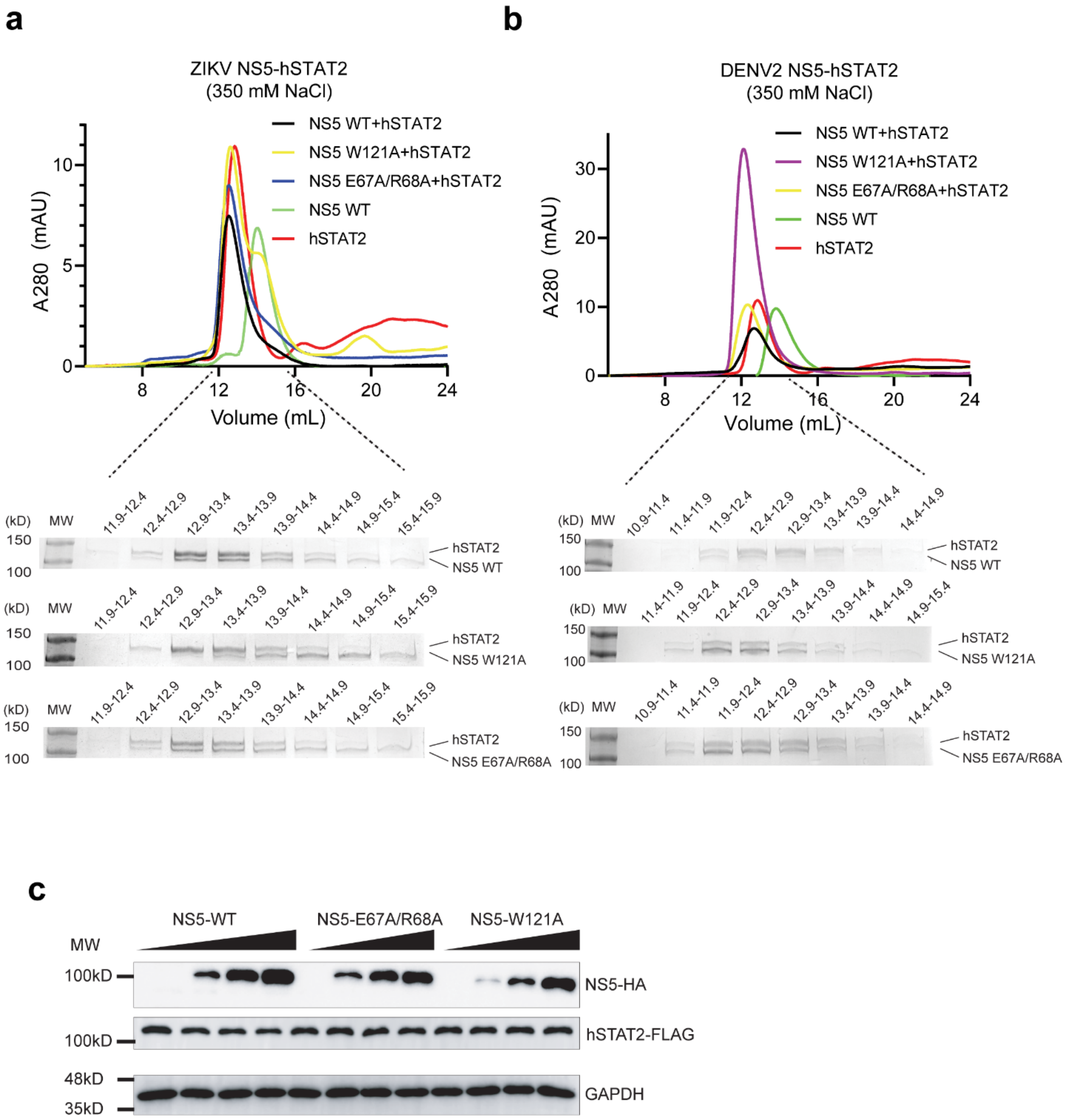

Supplementary Figure 7. Biochemical and cellular analyses of the interaction between flavivirus NS5 and hSTAT2. a, (top) Size exclusion chromatography analysis of ZIKV NS5, WT, W121A or E67A/R68A, mixed with hSTAT2 at 350 mM NaCl. The peak fractions were analyzed using SDS-PAGE (bottom). The elution profiles for apo forms of ZIKV NS5 and hSTAT2 were included as control. **b**, (top) Size exclusion chromatography analysis of ZIKV NS5, WT, W121A or E67A/R68A, mixed with hSTAT2 at 350 mM NaCl.

The peak fractions were analyzed using SDS-PAGE (bottom). The elution profiles for apo forms of DENV2 NS5 and hSTAT2 are included as control. **c**, Immunoblot analysis of 293T cells transfected with indicated plasmids encoding WT or conformation-specific mutant DENV2 NS5-HAs at increasing amounts using antibodies against HA, FLAG, and GAPDH. Related to Fig. 3j.

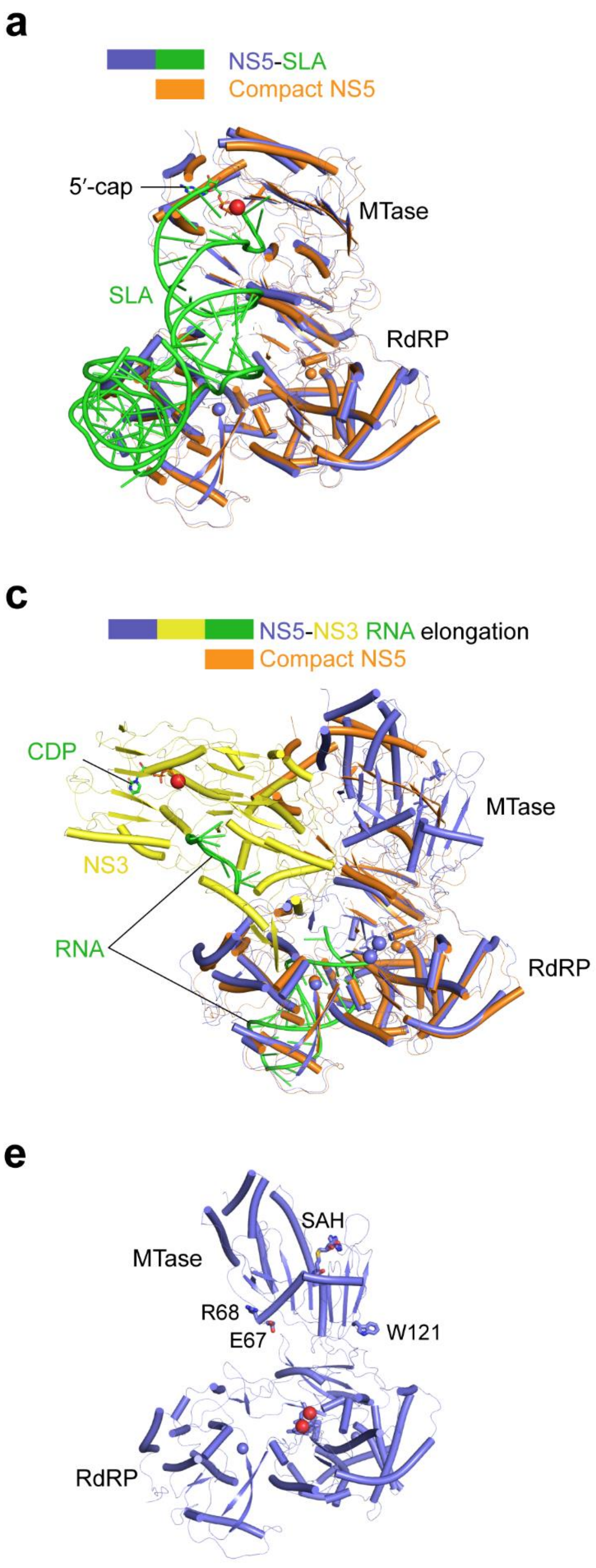
a
NS5-SLA
Compact NS5
5′-cap
MTase
SLA
RdRP
b
NS5-SLA
NS5-hSTAT2
5′-cap
MTase
hSTAT2
RdRP
SLA
c
NS5-NS3 RNA elongation
Compact NS5
CDP
MTase
NS3
RNA
RdRP
d
NS5-NS3 RNA elongation
NS5-hSTAT2
CDP
MTase
hSTAT2
NS3
RdRP
RNA
e
SAH
MTase
R68
E67
W121
RdRP

Supplementary Fig. 8. Structural comparison of the DENV2 NS5–hSTAT2 complex, compact form of apo DENV2 NS5, and the RNA replication complexes of DENV3 NS5. a, Structural superposition between the DENV3 NS5–SLA complex (PDB 8GZP) and the compact conformation of DENV2 NS5 (PDB 6KR3). The zinc ions are shown in sphere representation, colored slate in DENV3 NS5 and orange in DENV2 NS5. The Mg^{2+} ion is shown as a red sphere. The GDP bound to DENV3 NS5 is shown stick representation. **b,** Structural superposition between the DENV3 NS5-SLA complex (PDB 8GZP) and the DENV2 NS5–hSTAT2 complex. The zinc ions are shown in sphere representation, colored slate in DENV3 NS5 and aquamarine in DENV2 NS5. The Mg^{2+} ion is shown as a red sphere. The GDP bound to DENV3 NS5 is shown stick representation. **c,** Structural superposition between the DENV3 NS5–NS3–RNA elongation complex (PDB 8GZR) and the compact conformation of DENV2 NS5 (PDB 6KR3). The zinc ions are shown in sphere representation, colored slate in DENV3 NS5 and orange in DENV2 NS5. The Mn^{2+} ions are shown as red spheres. The CDP bound to DENV3 NS3 is shown stick representation. **d,** Structural superposition between the DENV3 NS5-NS3-RNA elongation complex (PDB 8GZR) and the DENV2 NS5–hSTAT2 complex. The zinc ions are shown in sphere representation, colored slate in DENV3 NS5 and aquamarine in DENV2 NS5. The Mn^{2+} ions are shown as red spheres. The CDP bound to DENV3 NS3 is shown stick representation. **e,** Inter-domain contact of DENV3 NS5 in the DENV3 NS5–NS3–RNA elongation complex (PDB 8GZR). Residues E67, R68 and W121, which mediate the inter-domain contacts of the compact or extended conformation of the NS5 protein, are not involved in the inter-domain contact in the NS5–NS3–RNA elongation complex. The zinc ions are shown in sphere representation. The Mn^{2+} ions are shown as red spheres. DENV3 NS3 and RNA molecules are not shown for clarity.

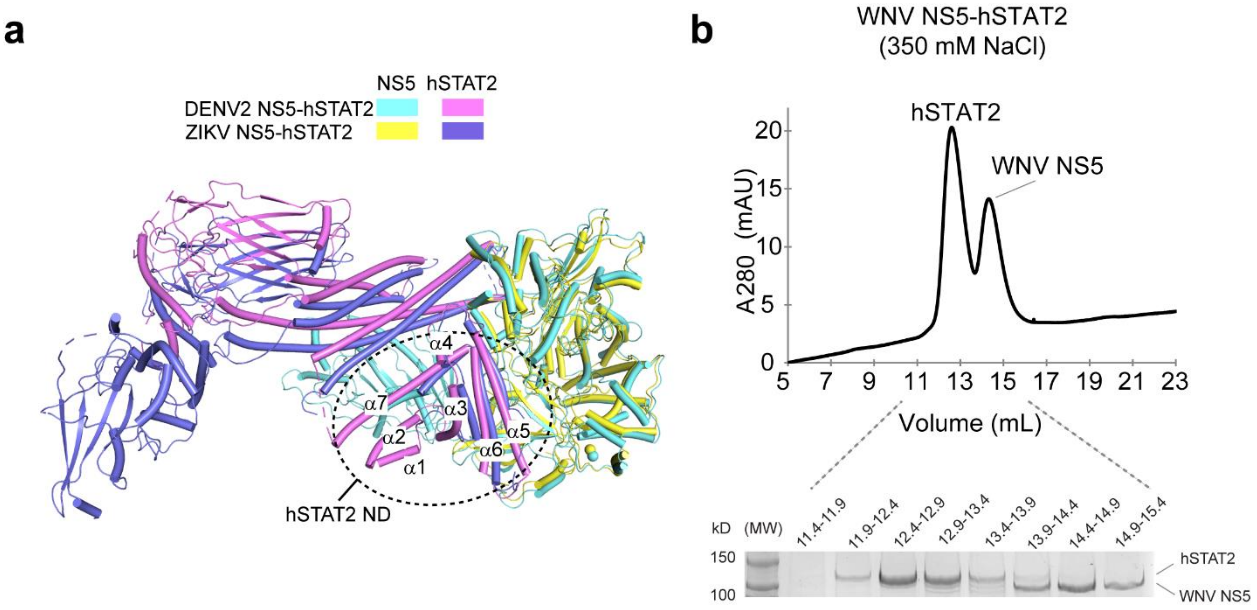

Supplementary Figure 9. Comparative analysis of flavivirus NS5–hSTAT2 interactions. a, Alignment of the cryo-EM structure of full-length DENV2 NS5–hSTAT2 complex with the crystal structure of ZIKV RdRP–hSTAT2 complex (PDB 6UX2). The α-helices for the hSTAT2 ND are labeled. Note that the hSTAT2 ND is fully defined in the DENV2 NS5–hSTAT2 complex, but only partially traced in the ZIKV RdRP–hSTAT2 complex. **b**, (top) Size exclusion chromatography analysis of WNV NS5 mixed with hSTAT2 at 350 mM NaCl. The peak fractions were analyzed using SDS-PAGE (bottom).

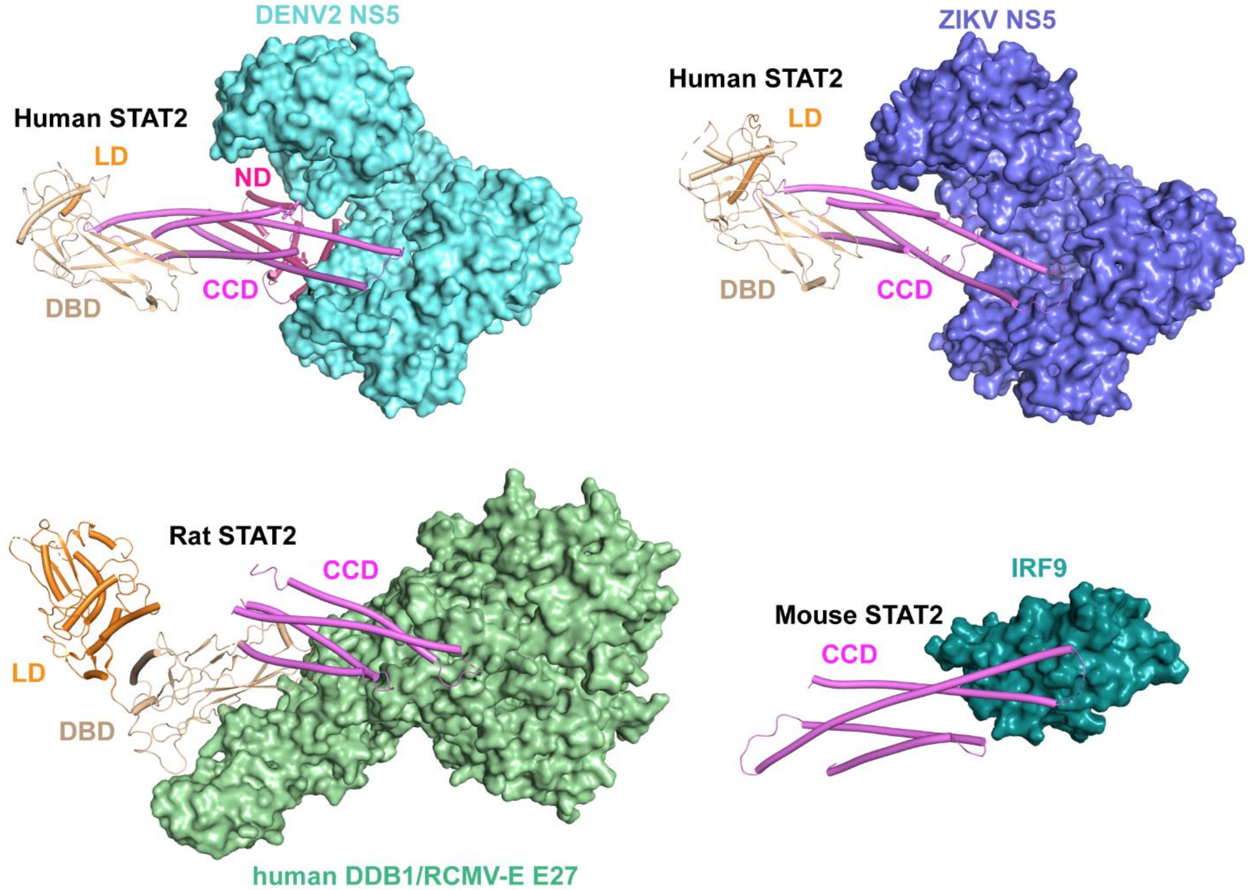

Supplementary Fig. 10. Structural comparison of STAT2 proteins (ribbon representation) in complex with DENV2 NS5 (surface representation), ZIKV NS5 (PDB 6WCZ, surface representation), human DDB1 and RCMV-E E27 (PDB 7ZNN, surface representation) and IRF9 (PDB 5OEN, surface representation).

For Figure 3h

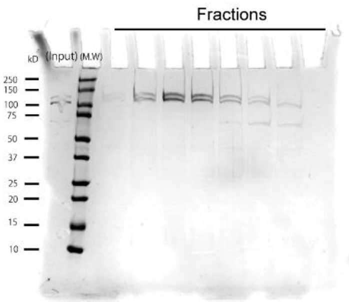

DENV2 NS5 WT + hSTAT2

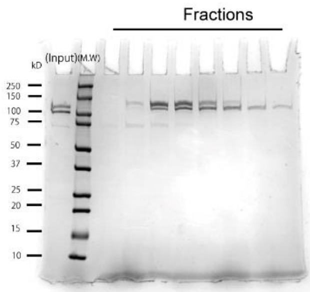

DENV2 NS5 W121A + hSTAT2

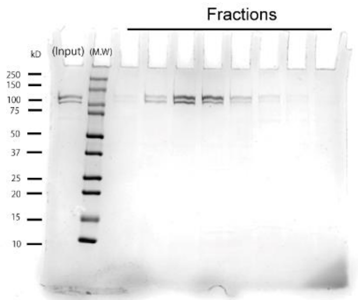

DENV2 NS5 E67A/R68A + hSTAT2

For Supplementary Figure 4g

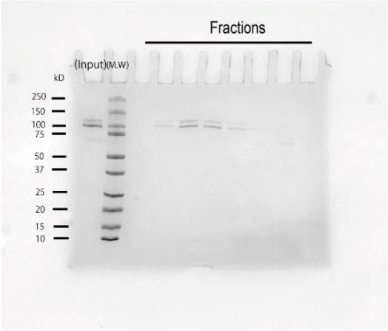

DENV2 NS5 WT + hSTAT2

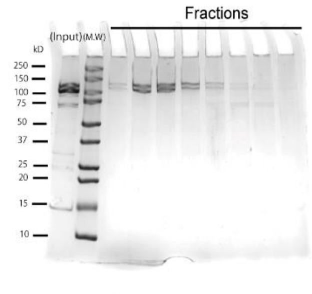

DENV2 NS5 P108G/H110A + hSTAT2

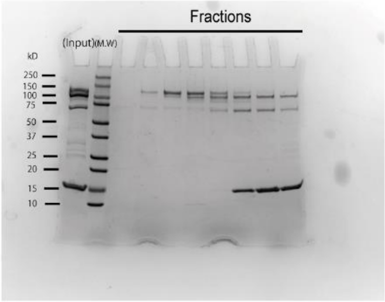

DENV2 NS5 P108G/H110A/P336G + hSTAT2

For Supplementary Figure 7a,b

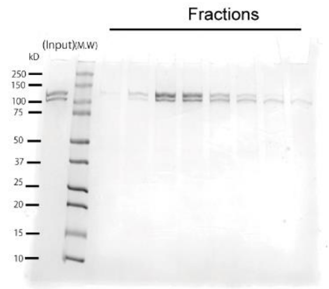

ZIKV NS5 WT + hSTAT2

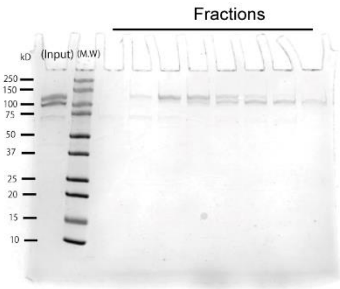

ZIKV NS5 W121A + hSTAT2

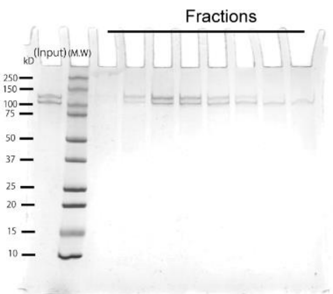

ZIKV NS5 E67A/R68A + hSTAT2

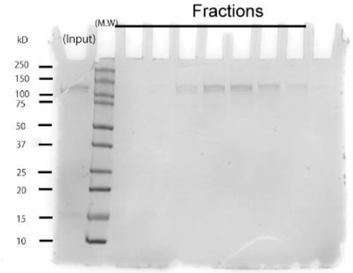

DENV2 NS5 WT + hSTAT2

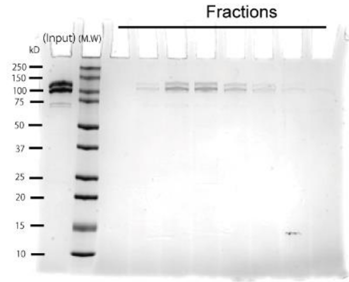

DENV2 NS5 W121A+ hSTAT2

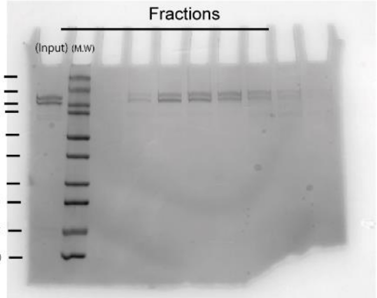

DENV2 NS5 E67A/R68A+ hSTAT2

For Supplementary Figure 9b

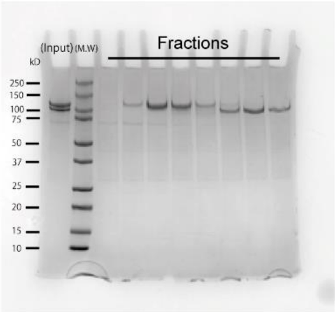

WNV NS5+ hSTAT2

For Figure 2d

IP: FLAG

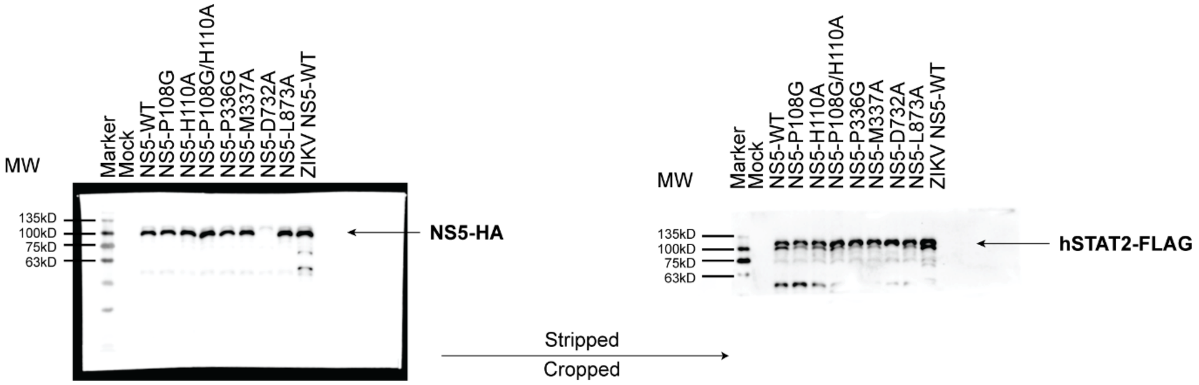

IP: HA

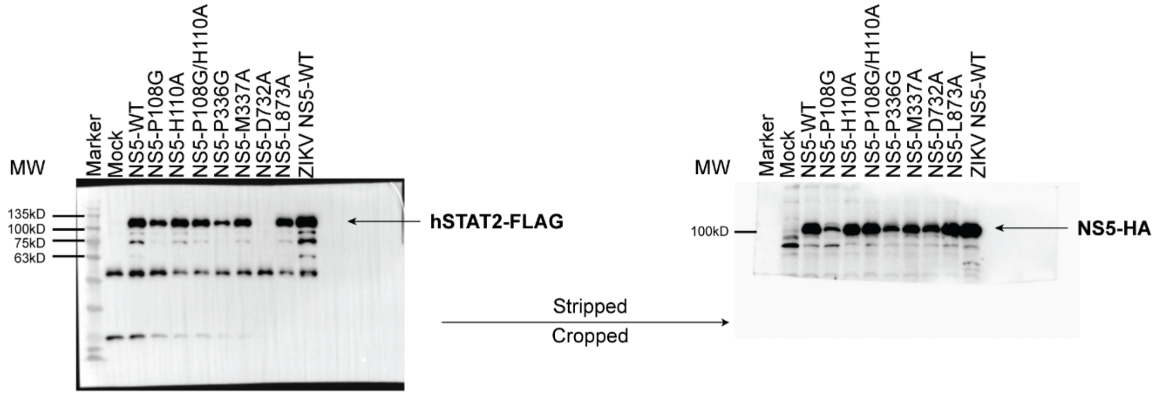

For Figure 2f

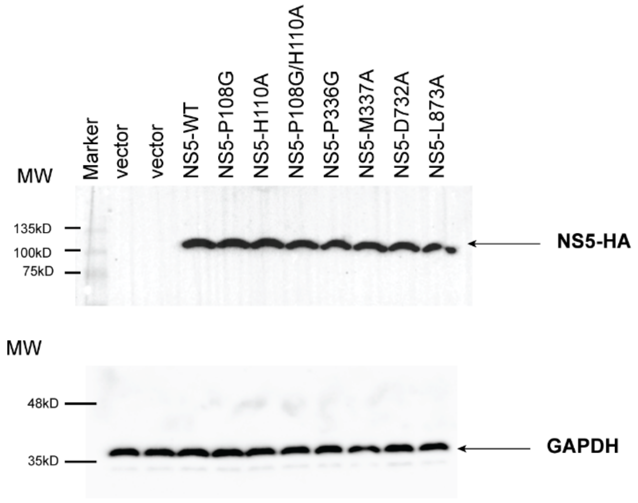

For Figure 3i

IP: FLAG

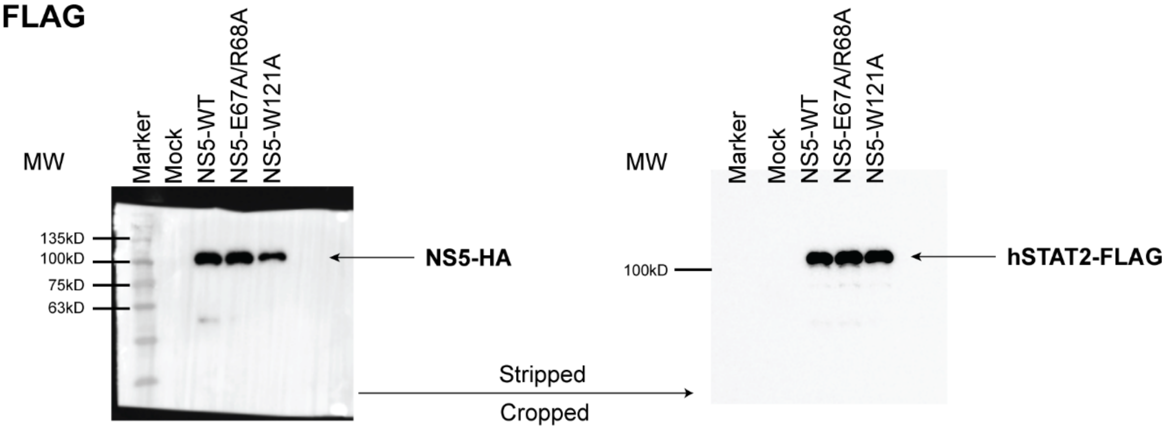

IP: HA

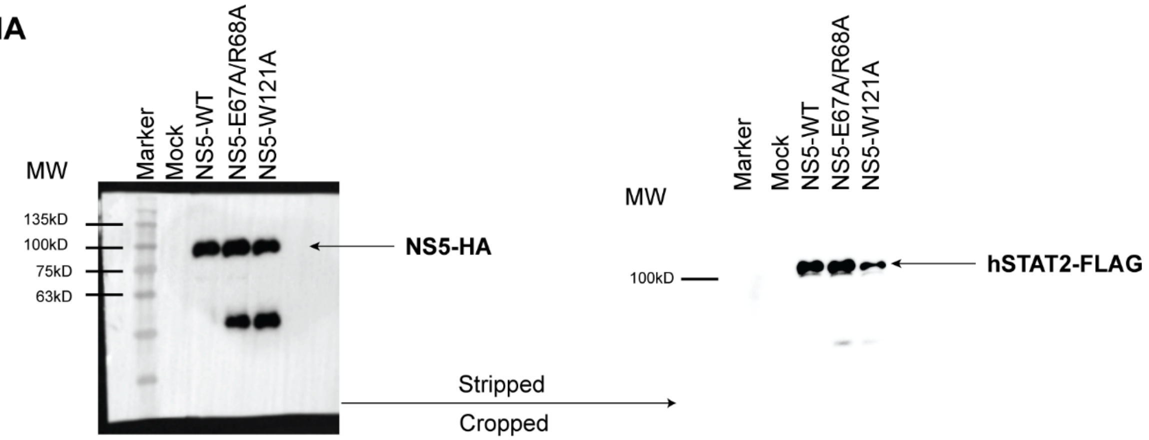

For Figure 3k

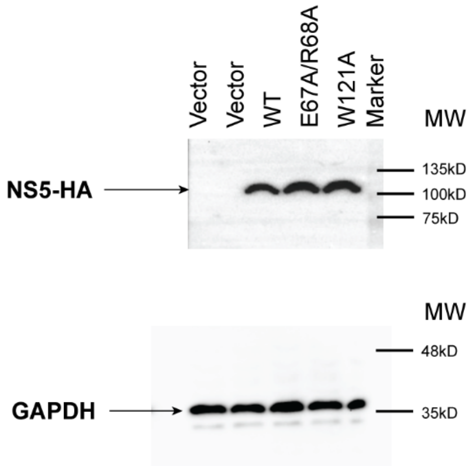

For Supplementary Figure 4a

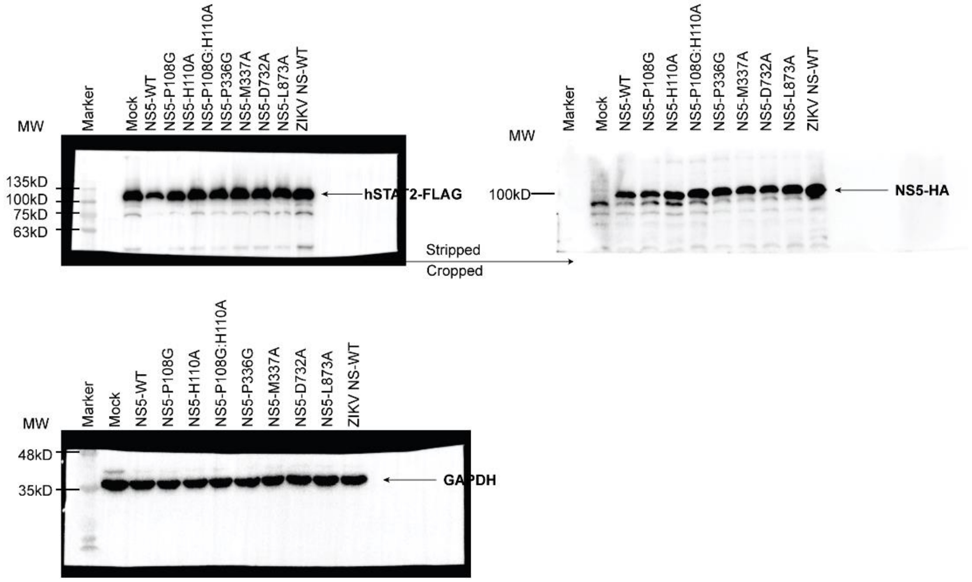

For Supplementary Figure 4d

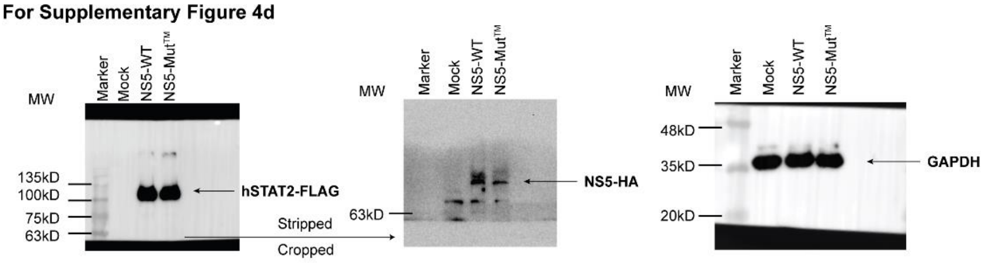

For Supplementary Figure 4e

IP: FLAG

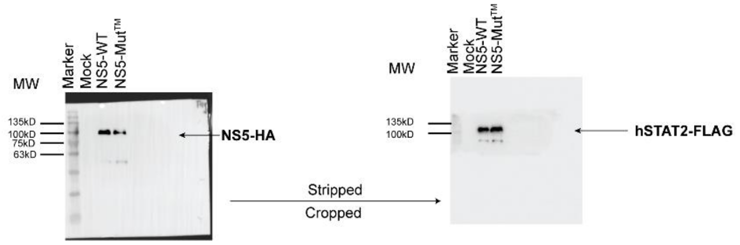

IP: HA

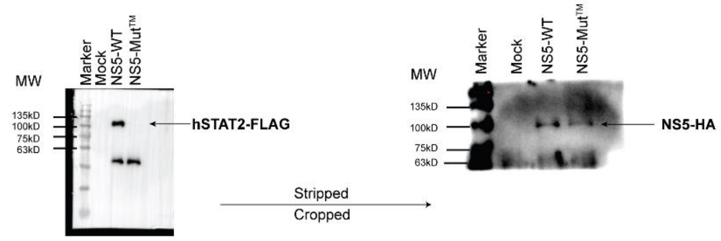

For Supplementary Figure 5a

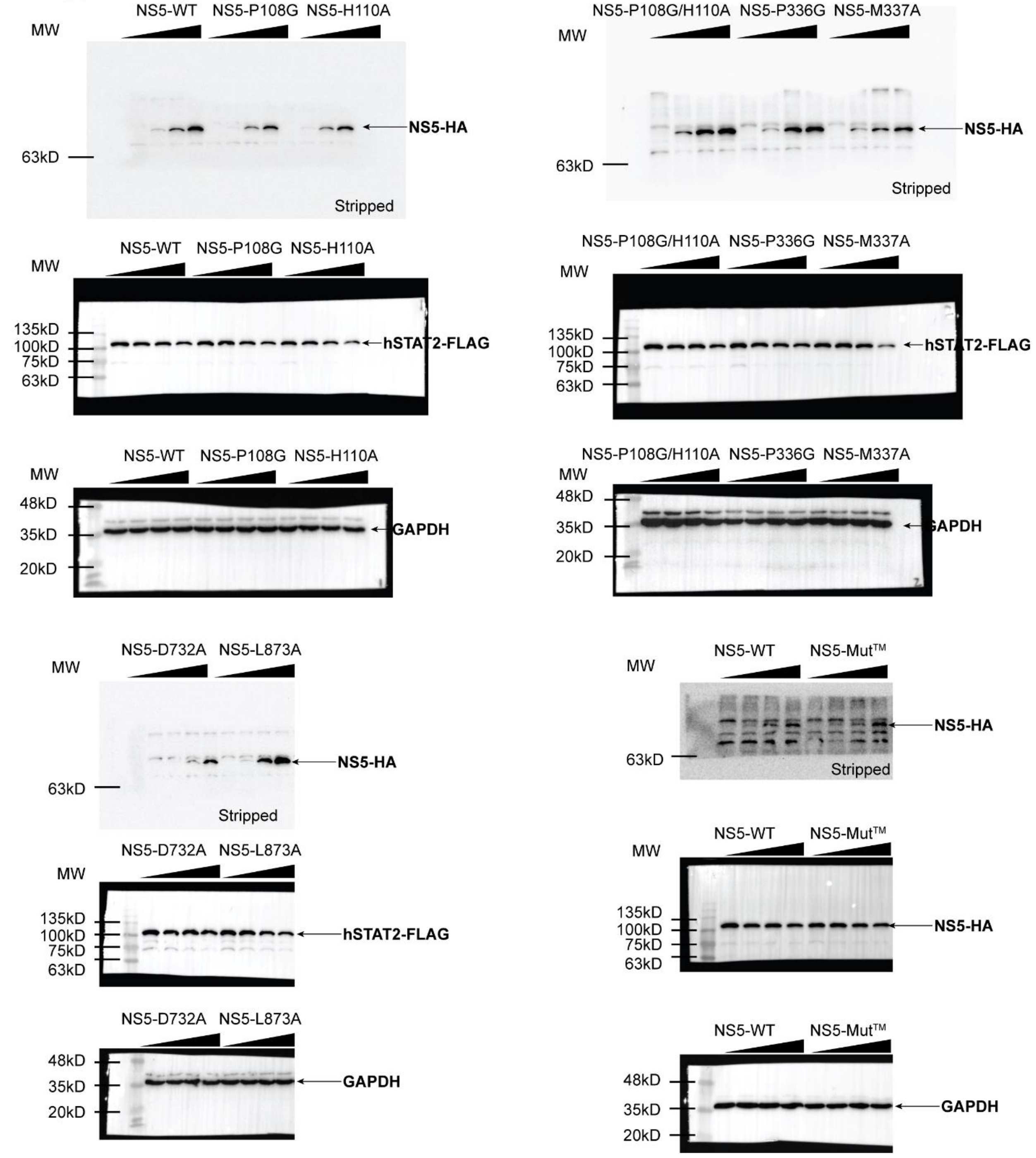

For Supplementary Figure 5c

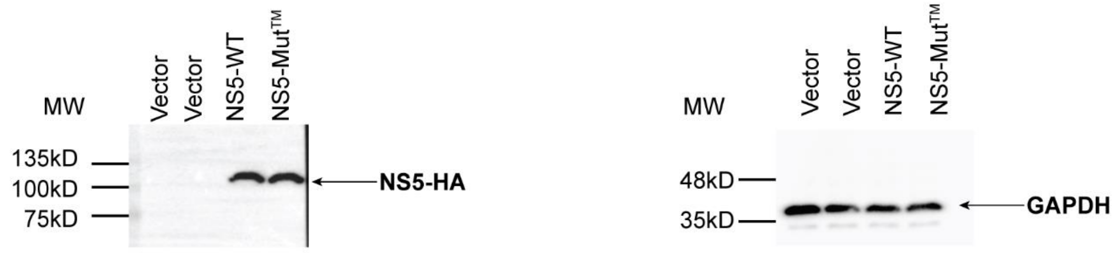

For Supplementary Figure 7c

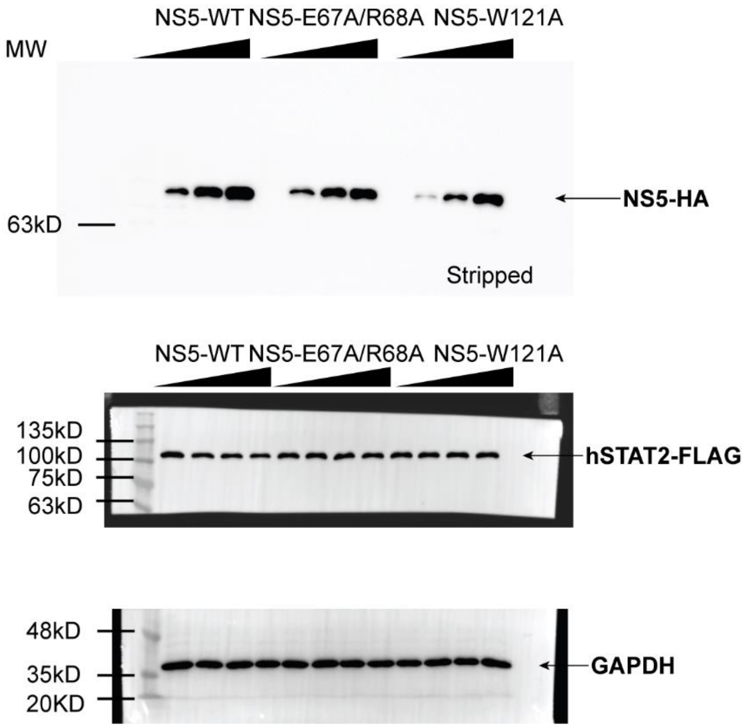

Supplementary Figure 11. Raw SDS-PAGE and western blot images used in this study.
