# Supplementary material for: A conformational selection mechanism of flavivirus NS5 for species-specific STAT2 inhibition
Source: Commun Biol. 2024 Jan 10;7:76. doi: 10.1038/s42003-024-05768-8 (PMC10776582; doi:10.1038/s42003-024-05768-8)
Supplement: Supplementary file 3 — Reporting Summary [file 42003_2024_5768_MOESM3_ESM.pdf]

nature portfolio

Corresponding author(s): Jikui Song, Rong Hai

Last updated by author(s): Dec 15, 2023

Reporting Summary

Nature Portfolio wishes to improve the reproducibility of the work that we publish. This form provides structure for consistency and transparency in reporting. For further information on Nature Portfolio policies, see our Editorial Policies and the Editorial Policy Checklist.

Statistics

For all statistical analyses, confirm that the following items are present in the figure legend, table legend, main text, or Methods section.

n/a	Confirmed	
☐	☒	The exact sample size (n) for each experimental group/condition, given as a discrete number and unit of measurement
☐	☒	A statement on whether measurements were taken from distinct samples or whether the same sample was measured repeatedly
☐	☒	The statistical test(s) used AND whether they are one- or two-sided<br>*Only common tests should be described solely by name; describe more complex techniques in the Methods section.*
☒	☐	A description of all covariates tested
☒	☐	A description of any assumptions or corrections, such as tests of normality and adjustment for multiple comparisons
☐	☒	A full description of the statistical parameters including central tendency (e.g. means) or other basic estimates (e.g. regression coefficient) AND variation (e.g. standard deviation) or associated estimates of uncertainty (e.g. confidence intervals)
☐	☒	For null hypothesis testing, the test statistic (e.g. F, t, r) with confidence intervals, effect sizes, degrees of freedom and P value noted<br>*Give P values as exact values whenever suitable.*
☒	☐	For Bayesian analysis, information on the choice of priors and Markov chain Monte Carlo settings
☒	☐	For hierarchical and complex designs, identification of the appropriate level for tests and full reporting of outcomes
☒	☐	Estimates of effect sizes (e.g. Cohen's d, Pearson's r), indicating how they were calculated

Our web collection on statistics for biologists contains articles on many of the points above.

Software and code

Policy information about availability of computer code

Data collection	The cryoEM data were collected from an FEI Titan Krios electron microscope at the National Cancer Institute (NCI).
Data analysis	cryoSPARC, Coot, Chimera X and Phenix softwares were used for data processing and structure determination.

For manuscripts utilizing custom algorithms or software that are central to the research but not yet described in published literature, software must be made available to editors and reviewers. We strongly encourage code deposition in a community repository (e.g. GitHub). See the Nature Portfolio guidelines for submitting code & software for further information.

Data

Policy information about availability of data

All manuscripts must include a data availability statement. This statement should provide the following information, where applicable:

- Accession codes, unique identifiers, or web links for publicly available datasets
- A description of any restrictions on data availability
- For clinical datasets or third party data, please ensure that the statement adheres to our policy

Coordinates and structure factors for the DENV2 NS5–hSTAT2 complex have been deposited in the Protein Data Bank with accession code 8T12 and 8T13. The cryo-EM density maps have been deposited to the EMDB and PDB under the accession numbers of EMD-40952 and EMD-40953, respectively. Source data are available with the paper online or Supplementary Fig. 11.

Research involving human participants, their data, or biological material

Policy information about studies with human participants or human data. See also policy information about sex, gender (identity/presentation), and sexual orientation and race, ethnicity and racism.

Reporting on sex and gender	N.A.
Reporting on race, ethnicity, or other socially relevant groupings	N.A.
Population characteristics	N.A.
Recruitment	N.A.
Ethics oversight	N.A.

Note that full information on the approval of the study protocol must also be provided in the manuscript.

Field-specific reporting

Please select the one below that is the best fit for your research. If you are not sure, read the appropriate sections before making your selection.

☒ Life sciences ☐ Behavioural & social sciences ☐ Ecological, evolutionary & environmental sciences

For a reference copy of the document with all sections, see nature.com/documents/nr-reporting-summary-flat.pdf

Life sciences study design

All studies must disclose on these points even when the disclosure is negative.

Sample size	Biochemical assays were completed using wild type or mutants of DENV NS5,ZIKV NS5, WNV NS5 or hSTAT2. Cellular assays were completed using wild type or mutant ZIKV NS5, DENV-2 NS5 or hSTAT2 proteins. The sample size is sufficient to delineate the mutational effects of NS5s and hSTAT2.
Data exclusions	No data exclusion.
Replication	For cellular assays, data are presented as the mean ± SD of at least 3 independent experiments. For cellular assays, at least 3 biological replicates were used and stated in figure legends. Statistical analysis was performed with Student's t test for comparing two sets of data with assumed normal distribution. A p value of less than 0.05 was considered to be significant.
Randomization	The assays performed in this study require a rational approach for activity comparison. Therefore, randomization is not applicable to our experimental set up.
Blinding	Blinding is not applicable to any biochemical or cellular assay performed in this study.

Reporting for specific materials, systems and methods

We require information from authors about some types of materials, experimental systems and methods used in many studies. Here, indicate whether each material, system or method listed is relevant to your study. If you are not sure if a list item applies to your research, read the appropriate section before selecting a response.

Materials & experimental systems

n/a	Involved in the study	
☐	☒	Antibodies
☐	☒	Eukaryotic cell lines
☒	☐	Palaeontology and archaeology
☒	☐	Animals and other organisms
☒	☐	Clinical data
☒	☐	Dual use research of concern
☒	☐	Plants

Methods

n/a	Involved in the study	
☒	☐	ChIP-seq
☒	☐	Flow cytometry
☒	☐	MRI-based neuroimaging

Antibodies

Antibodies used	Antibodies used for immunoblotting include anti-Flag (Sigma, F3165), anti-HA (Proteintech, Catalog #26183), anti-GAPDH (Proteintech, catalog no. 60004) and goat anti-mouse IgG (H+L) HRP-linked secondary antibody (Genesee Scientific, catalog no. 20-304).
Validation	They were validated by the manufacturers.

Eukaryotic cell lines

Policy information about cell lines and Sex and Gender in Research

Cell line source(s)	The source of each eukaryotic cell line was described in the section of "Plasmids and transfections".
Authentication	Authentication of cell line identity, including that of parental and derived lines, was ensured by using the genetic signature profiling analysis and observed for cell morphology routinely.
Mycoplasma contamination	All cells were tested mycoplasma negative using the PlasmoTest™ kit (InvivoGen) following the standard protocol.
Commonly misidentified lines (See ICLAC register)	N.A.

Plants

Seed stocks	N.A.
Novel plant genotypes	N.A.
Authentication	N.A.
